# Graphene Quantum Dots for Cell Proliferation, Nucleus Imaging, and Photoluminescent Sensing Applications

**DOI:** 10.1038/s41598-017-16025-w

**Published:** 2017-11-20

**Authors:** Mukesh Kumar Kumawat, Mukeshchand Thakur, Raju B. Gurung, Rohit Srivastava

**Affiliations:** Department of Biosciences and Bioengineering, Indian Institute of Technology Bombay, Mumbai, 400076 India

## Abstract

We report a simple one-pot microwave assisted “green synthesis” of Graphene Quantum Dots (GQDs) using grape seed extract as a green therapeutic carbon source. These GQDs readily self-assemble, hereafter referred to as “self-assembled” GQDs (sGQDs) in the aqueous medium. The sGQDs enter via caveolae and clathrin-mediated endocytosis and target themselves into cell nucleus within 6–8 h without additional assistance of external capping/targeting agent. The tendency to self-localize themselves into cell nucleus also remains consistent in different cell lines such as L929, HT-1080, MIA PaCa-2, HeLa, and MG-63 cells, thereby serving as a nucleus labelling agent. Furthermore, the sGQDs are highly biocompatible and act as an enhancer in cell proliferation in mouse fibroblasts as confirmed by *in vitro* wound scratch assay and cell cycle analysis. Also, photoluminescence property of sGQDs (lifetime circa (ca.) 10 ns) was used for optical pH sensing application. The sGQDs show linear, cyclic and reversible trend in its fluorescence intensity between pH 3 and pH 10 (response time: ~1 min, sensitivity −49.96 ± 3.5 mV/pH) thereby serving as a good pH sensing agent. A simple, cost-effective, scalable and green synthetic approach based sGQDs can be used to develop selective organelle labelling, nucleus targeting in theranostics, and optical sensing probes.

## Introduction

Graphene Quantum Dots (GQDs) are fluorescent carbon-based nanomaterials similar to graphene oxide (GO), an oxidized graphitic derivative, regarding structural and physical properties although they differ in size (<10 nm)^1,2^. Complicated synthetic procedures, complex surface chemistry, cellular toxicity, poor solubility, poor cellular uptake, and larger sizes limit the employment of carbon nanomaterials in the biological field^3–8^. To abate the above limitations, the GQDs (unlike other carbon nanomaterials) come with some advantages such as ease of their synthesis, alternative green synthetic procedures, ultra-small size, non-toxicity and excellent aqueous solubility^9,10^. Briefly, the GQDs can be fabricated using a top-down and bottom-up approaches^11^, recently, the green-chemistry-based approach has drawn a significant attention due to scalability, cost-effective non-toxic precursors, ease of synthesis, and competitive yield^9,10,12^. Naturally occurring plant materials^9,10,13^, food wastes^12^, milk^14^ and even harmful bacteria^15^ have proved to be excellent sources of carbon for GQDs synthesis. Poor uptake and toxicity of carbon nanomaterials are governed by physical parameters such as size, dimensions, surface functionalization, and solubility that curtails the possibilities of carrying out their intracellular behavior study and congenital biomedical applications^1,7,16^. However, few reports show that graphene-based composites have significant potential for wound healing^17^, cell adhesion^18^, and vasculogenesis^19^. The GQDs being nano-sized, highly biocompatible and highly dispersible in a biological medium, shown valuable catalytic properties which have been previously used for DNA cleavage^20^, and wound disinfection applications^21^. However, intracellular bio-applications of GQDs are still limited to drug delivery, bioimaging, and organelle labelling^22^.

**Figure 1:**
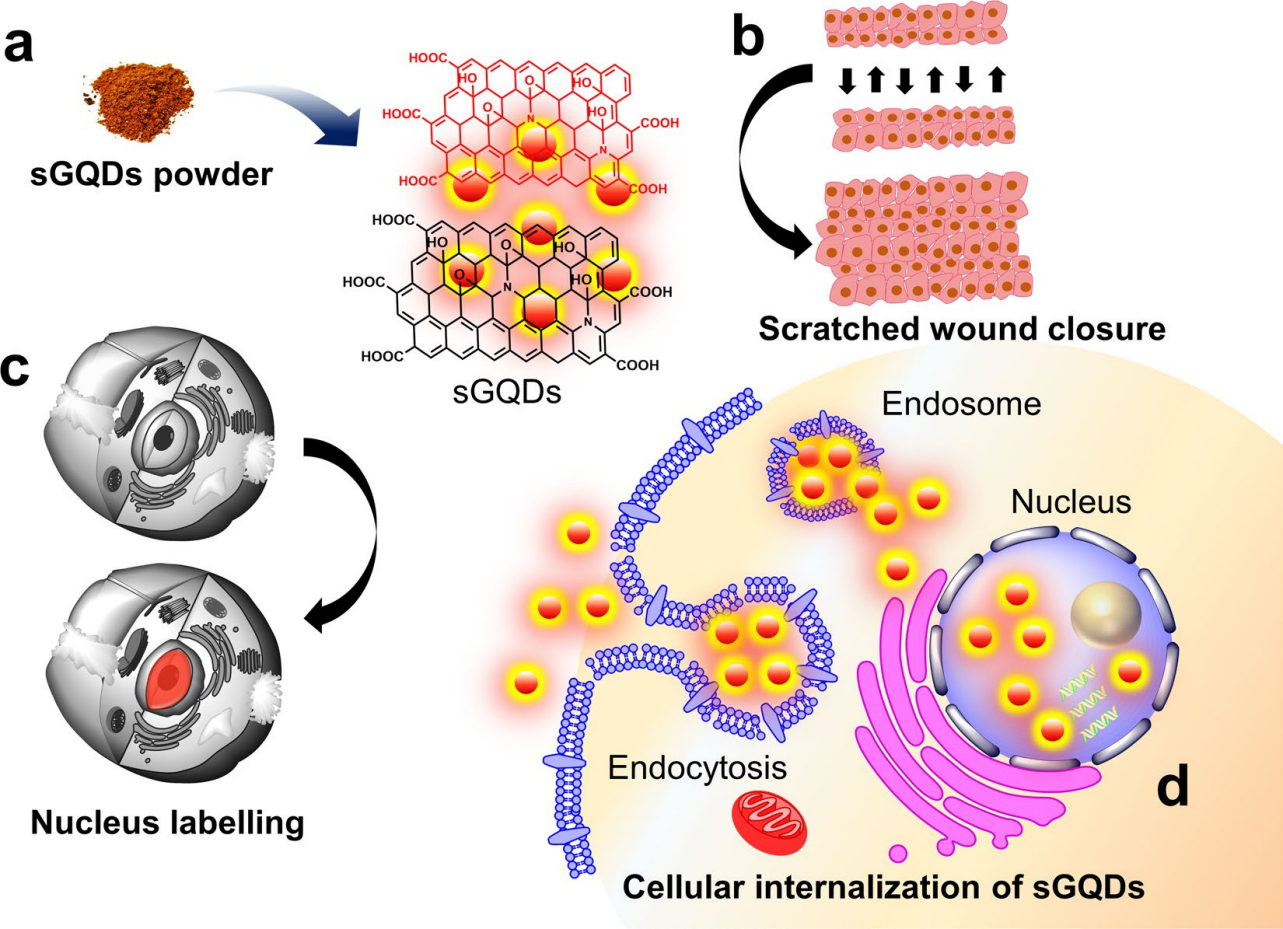
Schematic is showing (**a**) purified sGQDs powder, (**b**) rapid cell proliferation in the presence of sGQDs, (**c**) nucleus labelling using sGQDs and (**d**) cellular internalization of sGQDs and trafficking inside the nucleus.

The photoluminescence property of GQDs is widely explored in the field of bioimaging and sensing due to their narrow absorption and wide emission spectral characteristics. The other advantages include excellent optical properties such as tunable photoluminescence, excitation dependent and independent emission^2^ and resistance to photo-bleaching^11,22^. The tunable photoluminescence property is governed by choice of solvents, size, defect-states, the presence of heteroatoms (doping), and functional groups located on the surface of the GQDs^23,24^. Furthermore, unlike semiconductor-based quantum dots or organic dyes, the GQDs owe metal-free carbon precursor during synthesis and exhibit photostability, high *in vitro* as well as *in vivo* biocompatibility^13^. Cellular or sub-cellular labelling of the cells is of great interest in biology especially nucleus has been regarded as one of the main targets for cancer therapeutics^25^. Selective nucleus labelling can help to develop advanced and selective active-targeting drug delivery or gene delivery systems^25,26^. However, these systems as discussed earlier suffer from serious limitations such as photobleaching in organic dyes or cytotoxicity related to semiconductor quantum dots. The GQDs-based system serves as a good alternative to nucleus staining due to non-photobleaching, multi-photon emission, and good cellular distribution^27,28^. Recently, some reports have demonstrated fluorescent carbon nanomaterial-based nucleus staining application although affecting cell morphology^15,29^. While these reports demonstrated cellular nucleus labelling holistically, we show selective nucleus self-targeting GQDs towards labelling of normal cells within a short time ca. 8 h. In this report, a green synthesis approach was employed for fabrication of self-assembled GQDs (sGQDs) using grape seed extract (GSE). Dietary supplements of commercially available GSE consist of polyphenols which can serve as a “Therapeutic” carbon source for the green synthesis of the sGQDs. The extract is a good choice for the sGQDs synthesis because being a commercial product; it retains the consistency in the composition which otherwise varies in natural sources due to seasonal variation and geographical location. The GSE shows potent anti-oxidant, anti-microbial^30^, anti-ulcer^31^, and anti-cancer activity^32^. We demonstrate that the sGQDs fabricated using GSE, show rapid wound closure in an *in vitro* wound scratch assay. Also, the sGQDs internalize into the nucleus resulting in an accelerated cell proliferation (Fig. 1). Finally, based on a pH-responsive property of sGQDs, we show a linear fluorescence response to changes in narrow pH (pH 3–10) which can pave a way towards green-synthesis based cheaper and scalable sensing probes in future and have a huge potential in biomedical nanotechnology.

## Results and Discussion

### Physicochemical characterization

**Figure 2:**
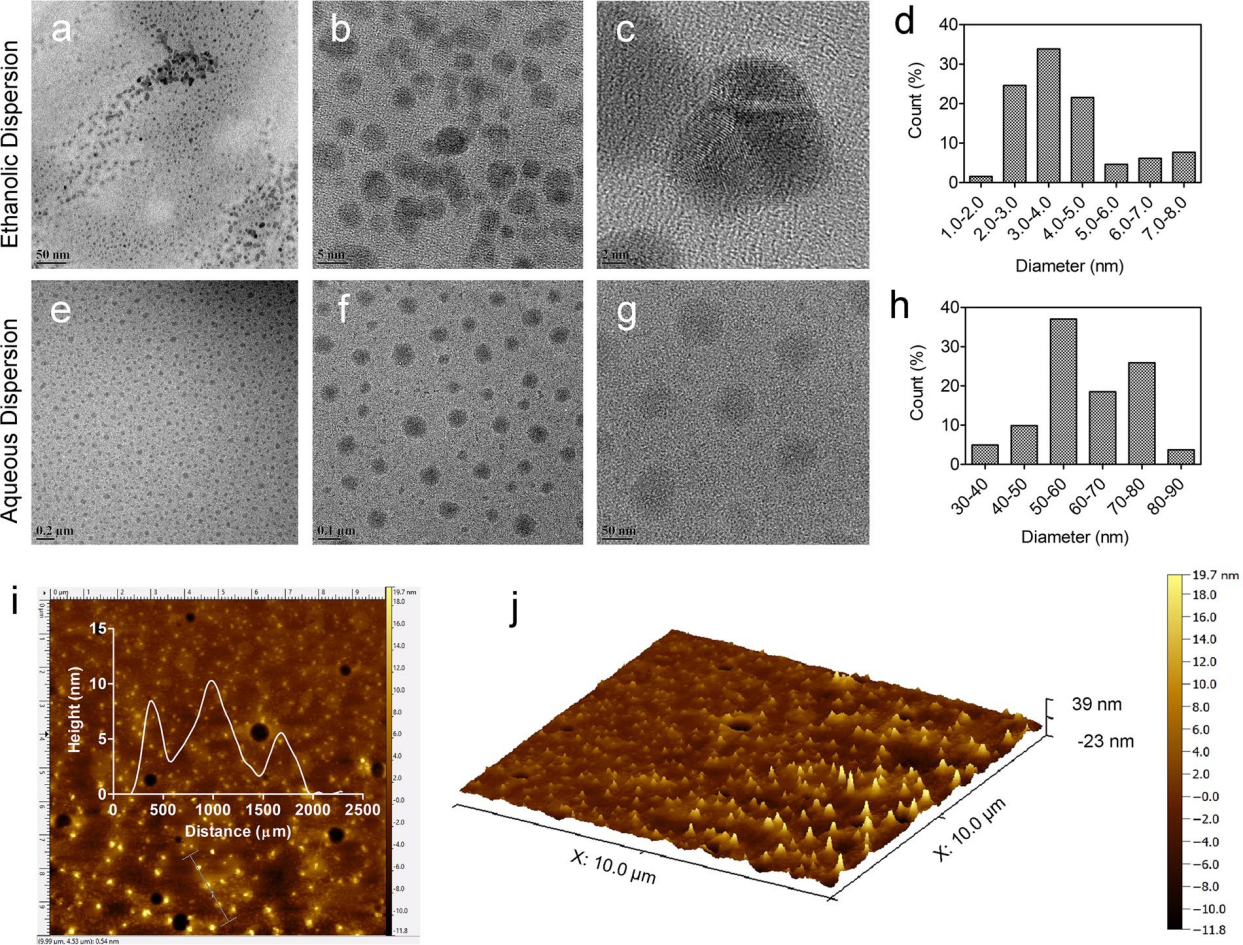
FEG-TEM and AFM micrographs showing two types of GQDs- the one dispersed in ethanol (non self-assembled) and an aqueous medium (self-assembled). (**a**–**d**) Uppermost panel, ethanolic dispersion show ultra-small GQDs ranging from 1–8 nm with most of them formed around 3.5 nm in diameter. (**e**–**h**) Middle panel shows the homogeneous distribution of sGQDs in an aqueous medium. Due to self-assembly, the size of sGQDs was found to be 50–60 nm in diameter (Calculated using Image **J**). (**i**–**j**) Bottom panel exhibits AFM micrographs of sGQDs dispersed in an aqueous medium which are up to 10 nm in height.

**Figure 3:**
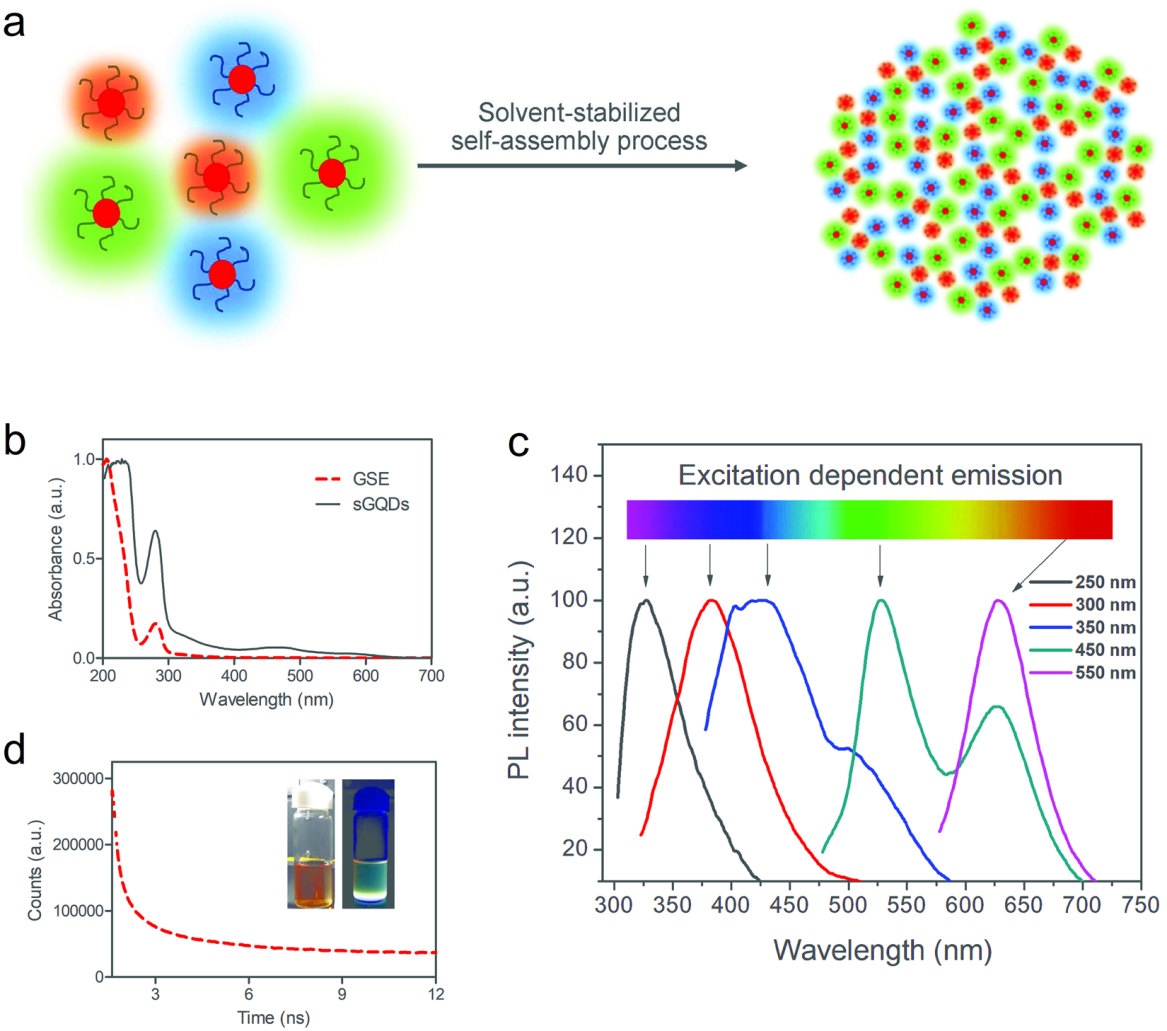
(**a**) Schematic showing transition of GQDs to sGQDs in an aqueous solution, (**b**) UV-Vis absorption spectrum of GSE and sGQDs, (**c**) photoluminescence emission spectra of sGQDs recorded at different excitation wavelengths, and (**d**) fluorescence lifetime of the sGQDs (10.04 ns) calculated using Time-Correlated Single Photon Counting instrument, inset showing images of sGQDs solution captured in daylight (left) and UV-light (right).

The sGQDs with a production yield of 53.6% and quantum yield of 31.79%(Supplementary Scheme S1) were obtained from an ethanolic extract of GSE powder(1.5 g) which is competitive to other green synthesized carbon sources^33^. Field Emission-Gun Transmission Electron Microscopic (FEG-TEM) analysis showed variable size distribution of GQDs based on their dispersion in different solvents (Fig. 2). When GQDs were purified and dispersed in ethanol, the size of the GQDs was relatively small i.e. 1–8 nm, whereas dispersion in aqueous medium produced sGQDs with size ~50–60 nm. The large sized sGQDs are a consequence of solvent stabilization resulting in the self-assembled units. Their self-assembly is driven by electrostatic interactions between superficial functional groups of nanomaterial and solvent^34,35^. The aqueous solution of sGQDs when subjected to sonication, showed an increment in fluorescence intensity due to the transient breaking of electrostatic interactions (Supplementary Fig. S1). The intermolecular arrangement of these nanostructures are due to π-π stacking, either overlapping or in a head-to-tail arrangement^36^. Further confirmation of head-to-tail arrangement can be elucidated by a broad peak in visible region around 470 nm (Fig. 3b) due to J-stacking and AFM image showing height profile where the width is increased while the height is few layers thick (Fig. 2i,j).

We studied optical and structural properties for further understanding the self-assembly process (Fig. 3a). A UV-Vis spectra shows strong absorption of as prepared sGQDs (100 µg mL^−1^) as compared to GSE (Fig. 3b). Strong characteristic absorption of sGQDs recorded around 220 nm and 290 nm due to π-π* transition arising out of sp^2^ aromatic domains and n- π* electronic transitions respectively^11,37^. The absorption decreases gradually towards higher wavelength and ends in the visible region showing excitation-dependent photoluminescence property with multiple excitation wavelengths (Fig. 3c). The absorption is concentration-dependent, and the FWHM increases with an increasing concentration of sGQDs (Supplementary Fig. S2)^36^. The multi-color emission property is due to emissive surface traps and oxygen-containing functional groups on sGQDs^38^. Photoluminescence lifetime was calculated at pH 7.0 and was found to be 10.04 ns (Fig. 3d).

**Figure 4:**
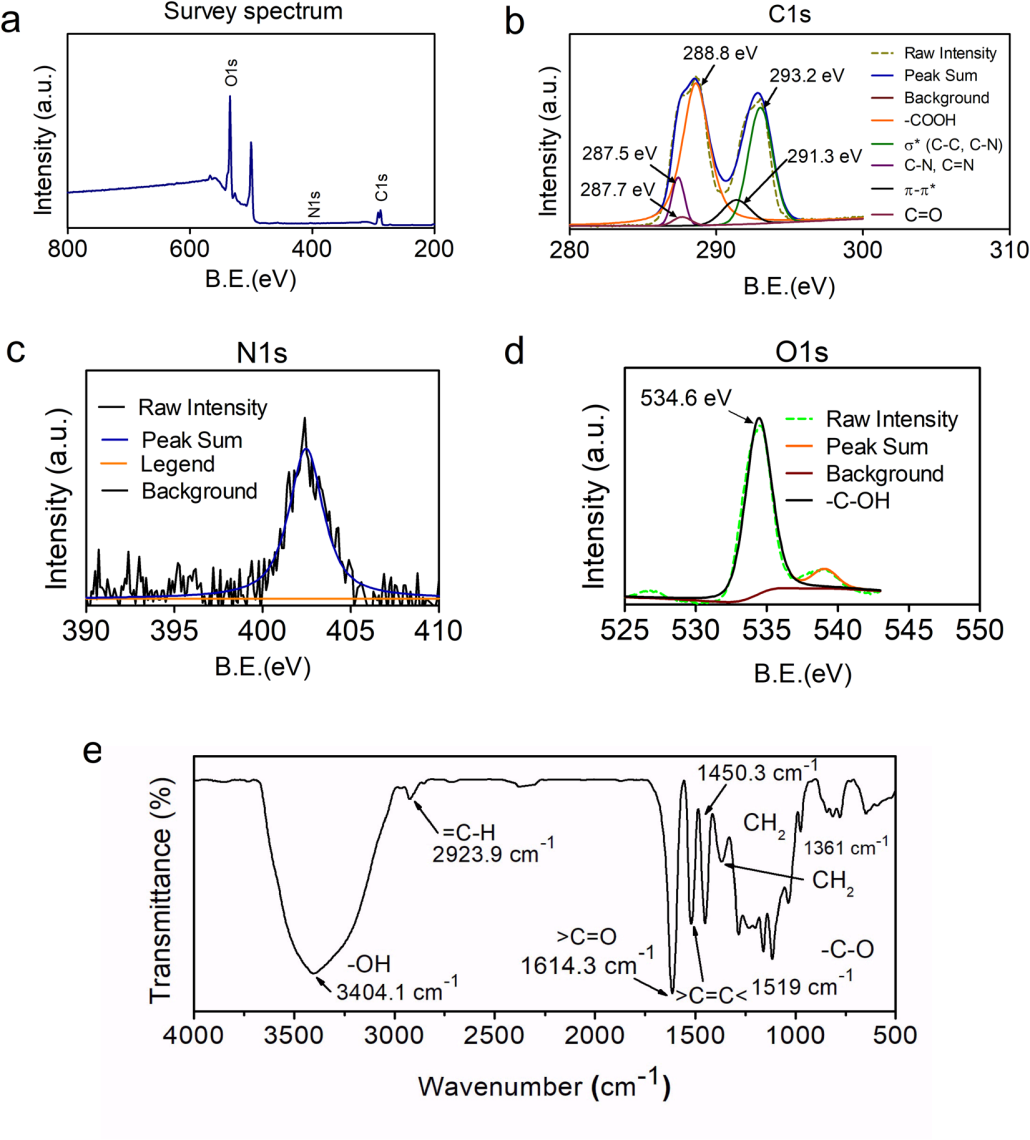
Physicochemical characterization of sGQDs showing (**a**) an XPS survey spectrum, and high-resolution XPS analysis for (**b**) C1s, (**c**) N1s, and (**d**) O1s spectrum, and (**e**) FTIR spectrum.

X-ray photoelectron spectroscopy (XPS analysis) of the sGQDs confirm the presence of C-C, C=O, C-O and C-N bonds in the structure of sGQDs (Fig. 4a). In detail, the C1s spectrum (Fig. 4b) can be deconvoluted in five peaks, such as, 287.5 eV (C-N), 287.7 eV (C=O), 288.0 eV (O-C=O), 291.3 eV (π-π*) and 293.2 eV (σ* C-C, C-N). The N1s (Fig. 4c) and O1s (Fig. 4d) spectra also confirm the presence of nitrogen and oxygen in the structure. FTIR spectrum (Fig. 4e) of the sGQDs, the peaks at 3404, 2923, 1614, 1519, 1320–1000 cm^−1^ are due to stretching vibrations in -OH, =C-H, >C=O, >C=C<, C-O bonds respectively^39,40^. The peaks at 1450 cm^−1^ and 1361 cm^−1^ are an indication of bending vibrations in >CH_2_ groups. The GSE is reportedly composed of polyphenols; predominantly proanthocyanidins like epicatechin, catechin, and gallic acid^41;^ the residual functional groups of these constituents resemble similar IR bands in the sGQDs. The XRD pattern (Supplementary Fig. S3) also supports the introduction of oxygen-containing functional groups which showed a broad peak at a 2θ value of 20.22° and greater interspacing distance of 0.438 nm than of graphite (0.34 nm), due to the incorporation of defects in the plane of the graphitic skeleton of the sGQDs^42^.

**Figure 5:**
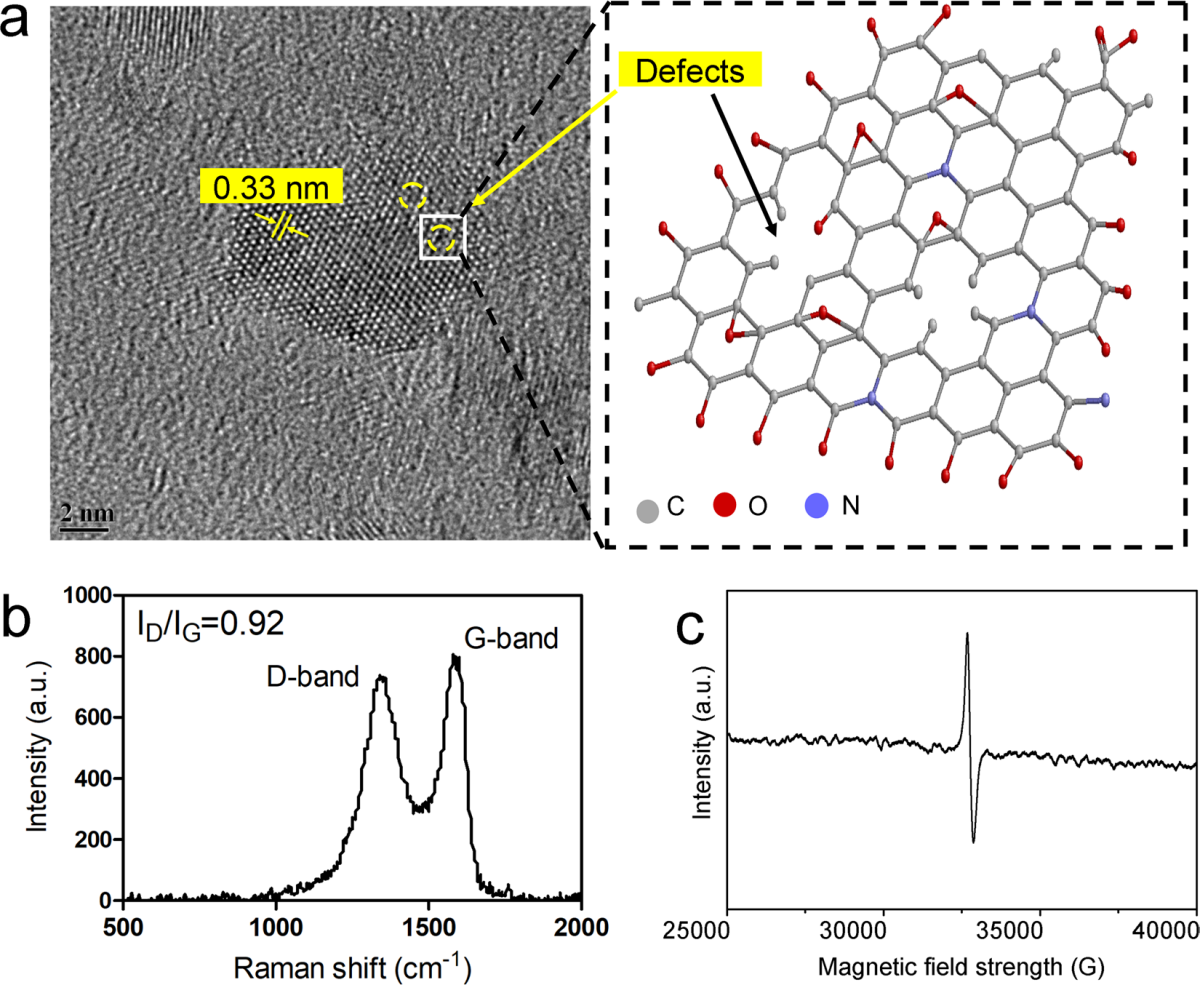
(**a**) An FEG-TEM micrograph showing lattice fringes with lattice spacing of 0.33 nm which corresponds to (006) diffraction plane of graphitic carbon (JCPDS 26–1076)^43^. The defects present in the sGQDs and proposed molecular structure model of the selected area. (**b**) Raman spectrum of GQDs showing graphitic as well as deformation peaks. (**c**) Electron Paramagnetic Resonance (EPR) spectrum of the sGQDs showing the presence of single electrons (g = 2.009, magnetic field = 335.824 mT) in the molecular structure of sGQDs.

The presence of functional groups also creates disorders (in forms of defects and differently hybridized atoms) in the graphitic carbon skeleton where both kinds of hybridized (sp^2^ and sp^3^) carbon atoms are prevalent. Defects in the skeleton of the sGQDs are visible at high resolution in FEG-TEM micrograph (200 keV, scale bar: 2 nm) based on which we propose a molecular structure model for better understanding (Fig. 5a). These results are consistent with the Raman spectrum of sGQDs confirming the disorder in the skeleton via characteristic defective or disorder peak (D-band) at 1330 cm^−1^ and a graphitic peak (G-band) observed at 1580 cm^−1^ (Fig. 5b). The presence of free electrons present in the structure was also confirmed by EPR spectrum at magnetic field value 335.8 mT (Fig. 5c).

### Biocompatibility, wound scratch assay and cell cycle analysis

**Figure 6:**
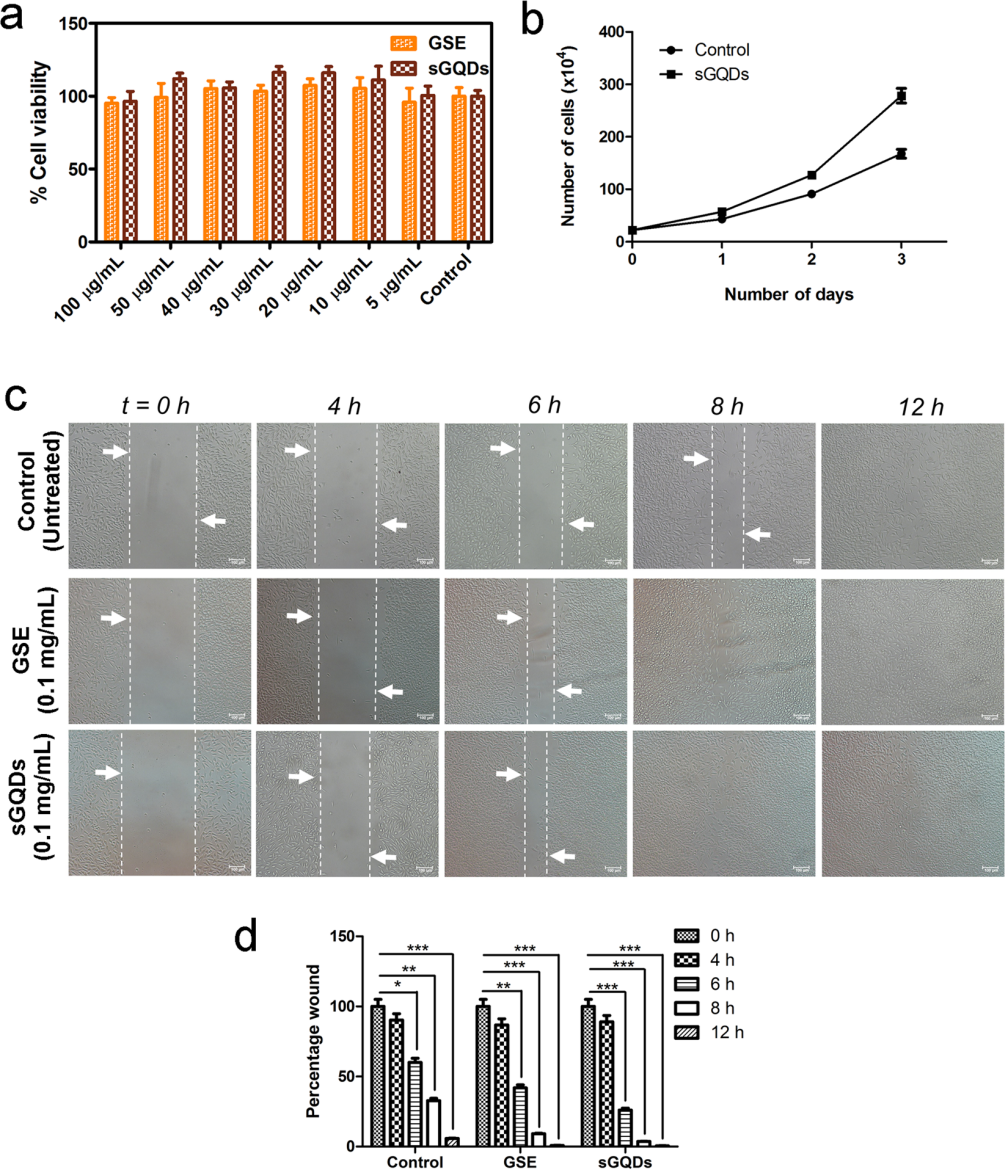
(**a**) Biocompatibility of GSE and sGQDs on fibroblasts L929 cells (n = 4). (**b**) Increasing number of a cell population after treatment with sGQDs compared to untreated control cells, data shown as mean ± standard deviation (n = 4), (**c**) a representative microscopic images showing *in vitro* cell proliferation-inducing property of GSE and sGQDs (Scale bar: 100 µm). (**d**) Comparative quantitative scratch wound closure (represented as percentage area of the wound) in GSE and sGQDs by *in vitro* scratch assay (n = 4, Two-way ANOVA, *P < 0.5, **P < 0.01, ***P < 0.001).

Cell viability assay was performed to analyze the cytotoxic effect of GSE and sGQDs on the normal L929 cells. Both GSE and sGQDs showed excellent biocompatibility at higher concentrations (upto 100 µg mL^−1^) after 24 h of treatment (Fig. 6a). Also, GSE and sGQDs do not show  reactive oxygen species (ROS) production indicating no cytotoxicity to the cells (Supplementary Fig. S4). Several reports have shown concerns about the cytotoxic effect of carbon nanomaterials on the cells *in vitro* ^44,45^. Recently, Qin *et al*. demonstrated that chemically fabricated GQDs cause apoptosis, autophagy, and inflammatory response^45^. Here, we demonstrate green-synthesized sGQDs with a proliferative activity *in vitro* as seen in Fig. 6b in comparison to untreated cells. *In vitro* scratch assay was performed using sGQDs, GSE and untreated cells as a control (Fig. 6c). The proliferation was enhanced in the presence of GSE and sGQDs and a complete closure of the scratched wound was observed (after 12 h), whereas untreated control cells did not show complete scratch wound closure within the same time interval. Figure 6d shows quantitative percentage scratch wound area with or without treatment for different time lapse. The sGQDs showed proliferative activity on L929 cells significantly as compared to control after 12 h (*P* < 0.001). Also, there was an increased scratch wound closure activity after 6 h in both GSE (*P* < 0.01) and sGQDs (*P* < 0.001). The sGQDs, however, showed potent proliferative activity in comparison to both control and GSE-treated cells (Fig. 6d).

**Figure 7:**
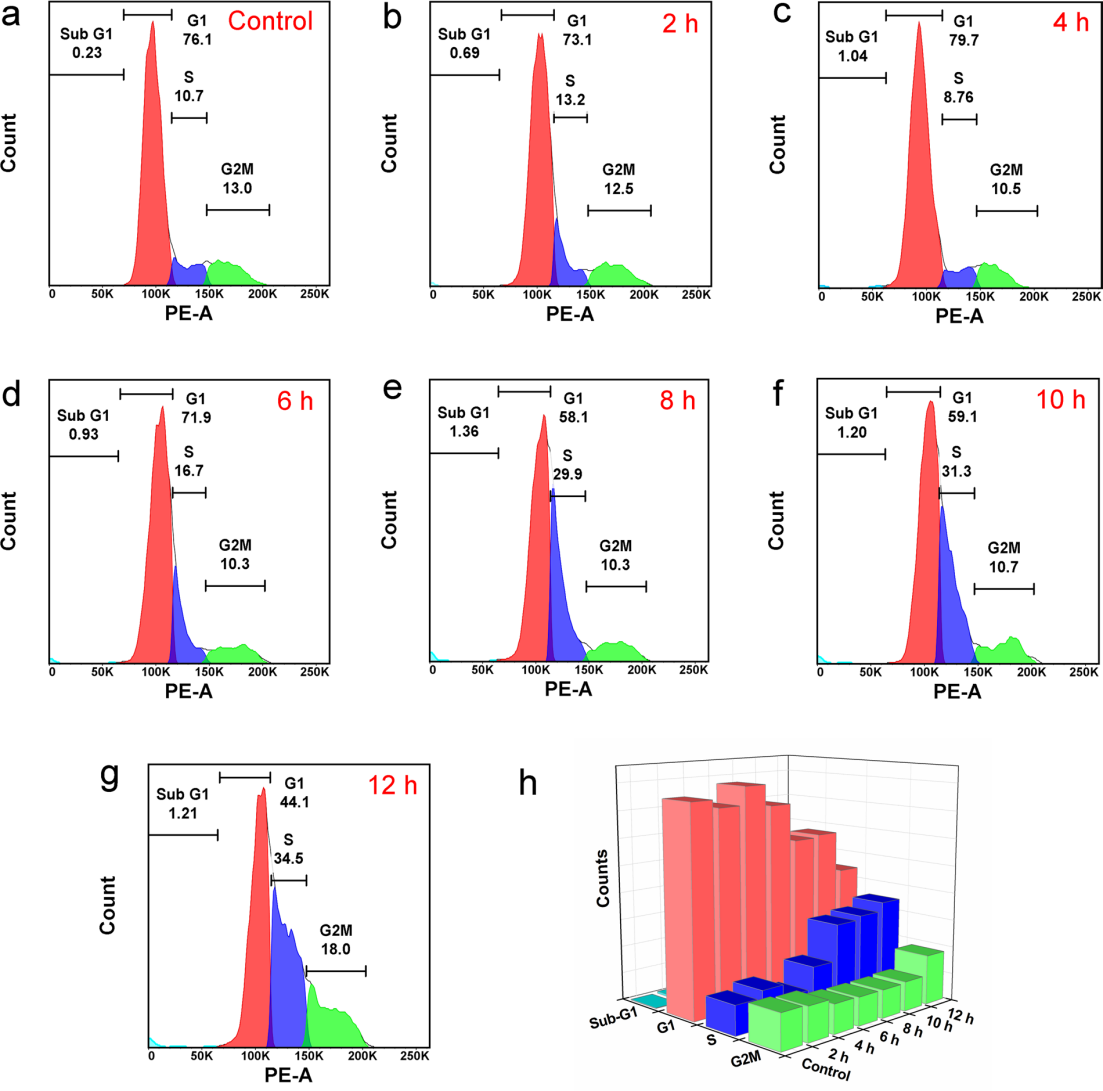
Differential cell cycle analysis of the actively dividing L929 cells after sGQDs treatment. Cell cycle analysis of (**a**) untreated cells (control cells) after 12 h. The images (**b**–**g**) are showing the cell cycle plot of the cells incubated with sGQDs for 2, 4, 6, 8, and 12 h, respectively. Image (**g**) shows a comparative 3D plot of the growth phases of cells.

The nucleus is a region where DNA replication occurs (in S-phase) followed by mitosis (in G_2_-phase). To understand the effect of proliferative activity on the cell division, we performed differential cell cycle study over L929 cells for different time periods (Fig. 7). A careful observation of cell cycle plots recorded at different time periods indicate the proliferating activity of sGQDs. A sudden rise in S-phase initially after 4 h from 8.76% to 16.7% and then to 29.9% after 6–8 h incubation of sGQDs (Fig. 7d,e) suggests the increased DNA content in the cells as compared to the control cells (Fig. 7h). The internalization of sGQDs was further studied by confocal laser scanning microscopy (CLSM) and flow cytometry.

### Nucleus labelling of cells using sGQDs

**Figure 8:**
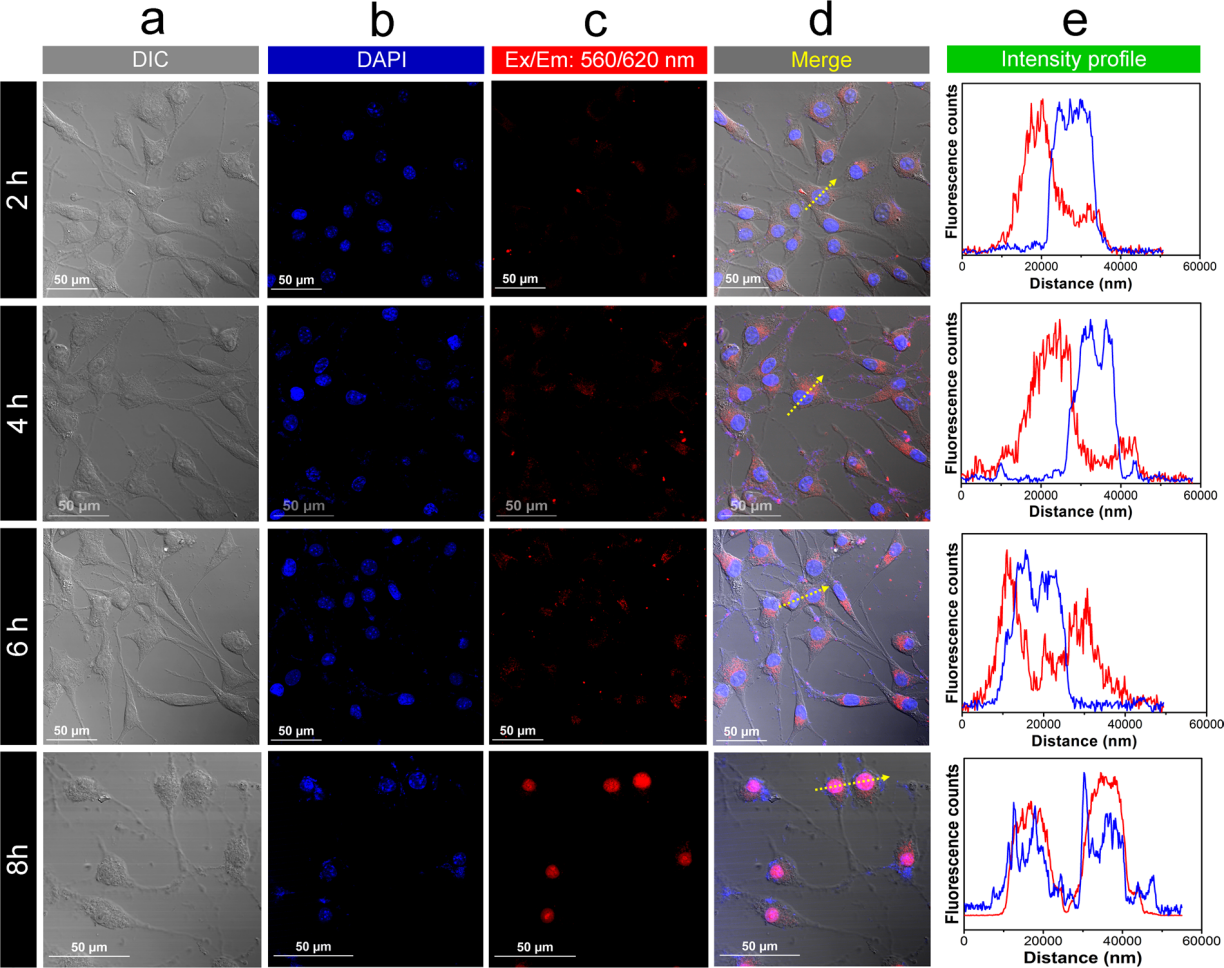
CLSM images are showing cellular intake and localization of sGQDs in the nuclei of L929 cells. (**a**) Differential Interference Contrast (DIC) image, (**b**) DAPI localization in the nuclei, (**c**) Red laser excitation is showing localization of the sGQDs from the cell cytoplasm to the nucleus, (**d**) Merged image of all channels, (**e**) Comparative fluorescence intensity plot of DAPI and sGQDs from the selected cells.

Figure 8 shows confocal laser microscopic images of fibroblasts co-incubated with sGQDs and DAPI. It is suggested that the sGQDs are readily taken up and are biocompatible with the L929 cells at 37 °C. The results corroborate with previous reports of GQDs^9,10^. In this report, we demonstrate nucleus staining of cells due to sGQDs localization inside the nucleus in relatively short time (8 h). In Fig. 8, the cells stained with DAPI show bright blue fluorescence indicating the nucleus region. Upon excitation with a red laser the cells showed bright red fluorescence from sGQDs inside the cells. The fluorescence intensity plot over the cells show the following two key information-

i. The fluorescence intensity of DAPI and sGQDs from the cells indicated by yellow arrow (Fig. 8, column d) and
ii. time-dependent shift in the fluorescence intensity of sGQDs from cytoplasm towards the nucleus as they traverse inside the nucleus.

The sGQDs self-directed themselves from cytoplasm towards nucleus within 8 h and localized themselves inside the nuclear region. It is demonstrated that fluorescence co-localisation of sGQDs and DAPI overlaps inside the cell nucleus meagrely at 6 h and completely after 8 h (Fig. 8, right panel). The sGQDs entered into the cells (within 2 h) and traversed towards the nucleus thereafter, till completely self-localising inside the nucleus (within 8 h), and same corroborate with the cell cycle results as discussed earlier. To further understand the intranuclear localization of sGQDs; three-dimensional (3D) localization inside the cell was studied (Supplementary Fig. S5 and S6). Similar results were observed when sGQDs were treated in different cell lines from various origins. The sGQDs entered the cells and self-localized themselves in the cell nucleus of HT-1080 (fibrosarcoma), MIA PaCa-2 (pancreatic cancer), HeLa (ovarian cancer), and MG-63 (osteosarcoma) cell lines, see Supplementary Fig. S7. The quantitative and 3D co-localization shows that the sGQDs co-localised with DAPI in the cell nucleus within 8 h. Thus, the sGQDs could be used for nucleus labelling of different cell lines. Since, very few nuclear labelling commercial dyes are available for live cell imaging, sGQDs can form a novel and cost-effective alternative imaging agent.

**Figure 9:**
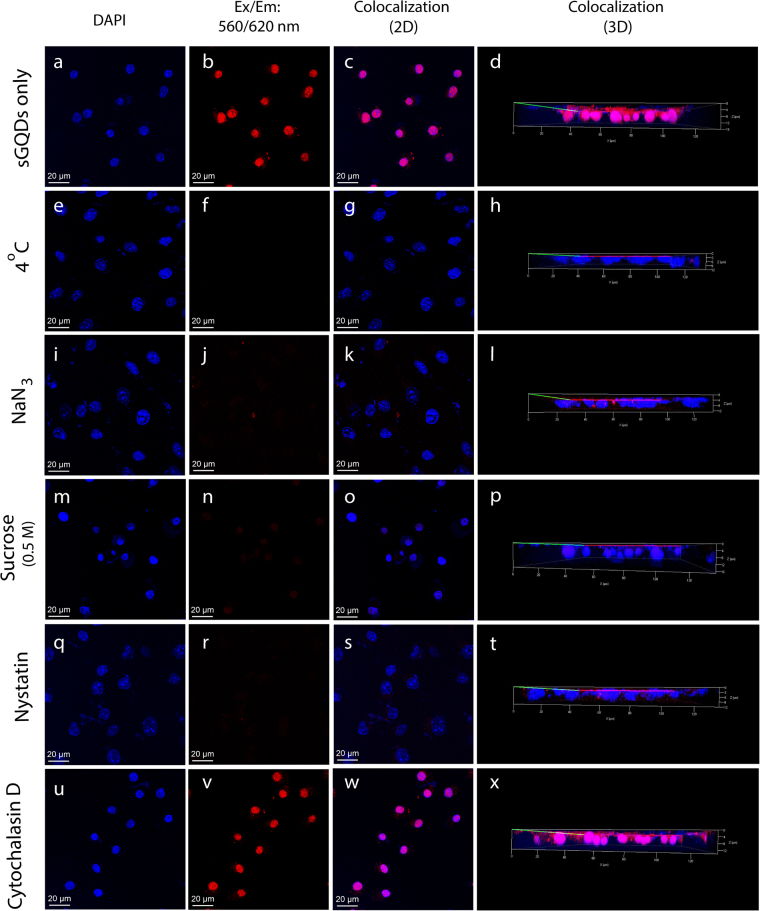
A CLSM imaging showing (**a**–**d**) qualitative cellular uptake of sGQDs only, and (**e**–**h**) sGQDs incubated at 4 °C or (**i**–**l**) at 37 °C with NaN_3_. Other L929 cells were treated with (**m**–**p**) hypertonic sucrose (0.5 M), (**q**–**t**) nystatin, and (**u**–**x**) cytochalasin D up to 8 h.

**Figure 10:**
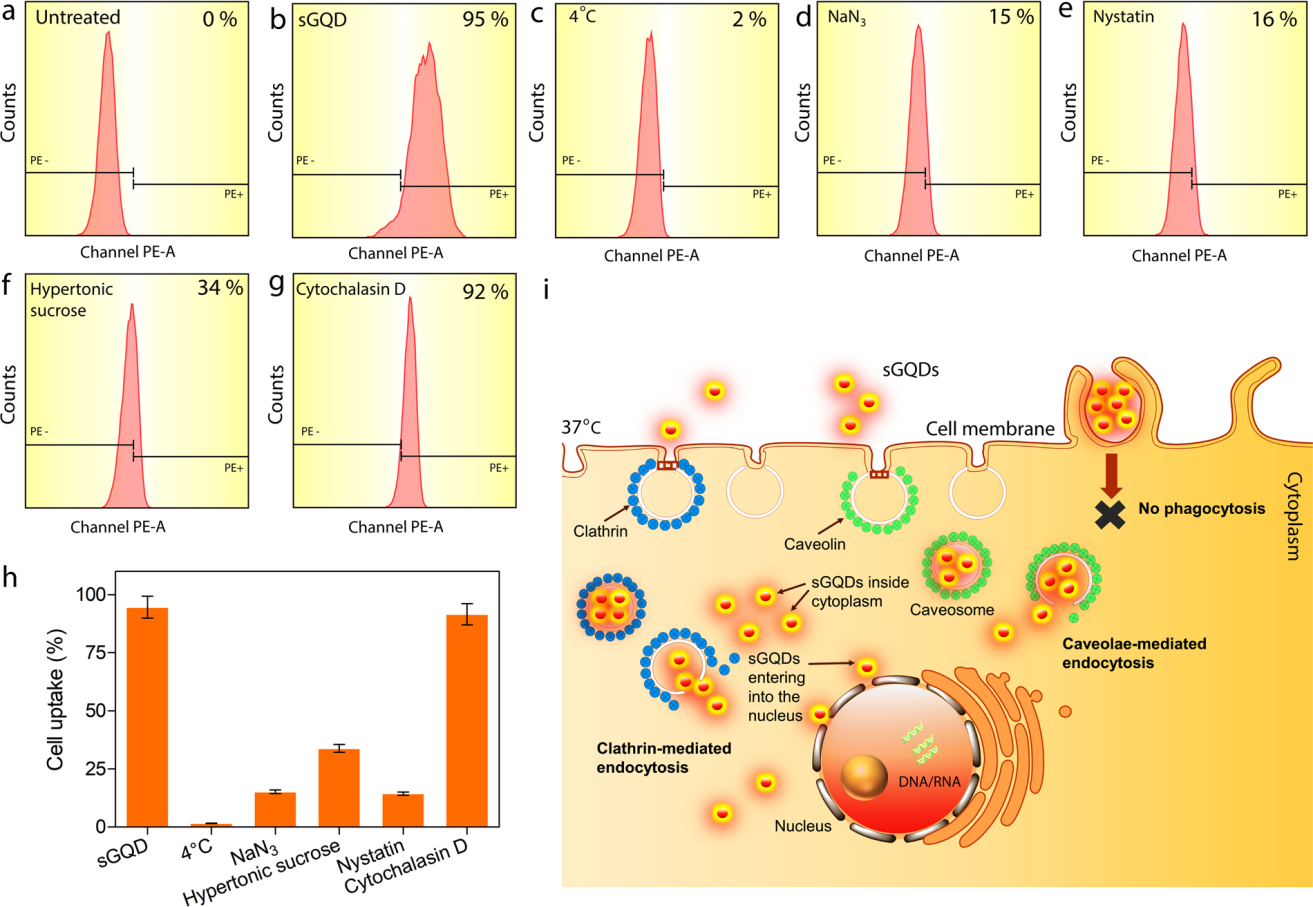
Cell uptake analysis of sGQDs using flow cytometry in L929 cells. For the quantification of cell uptake, (**a**) untreated cells were considered as a negative control, and (**b**) cells treated with sGQDs only were regarded as positive control. (**c**) For energy-dependent uptake analysis, the cells were incubated with sGQDs at 4 °C. or (**d**) cells were incubated with sGQDs and NaN_3_ at 37 °C. (**e**) For other cell uptake analysis, the cells were co-incubated with sGQDs and nystatin, (**f**) hypertonic sucrose solution (0.5 M), and (**g**) cytochalasin D, and (**h**) the quantification of sGQDs. (**i**) Schematic showing cell uptake mechanisms of sGQDs and their localization in the cell nucleus.

**Figure 11:**
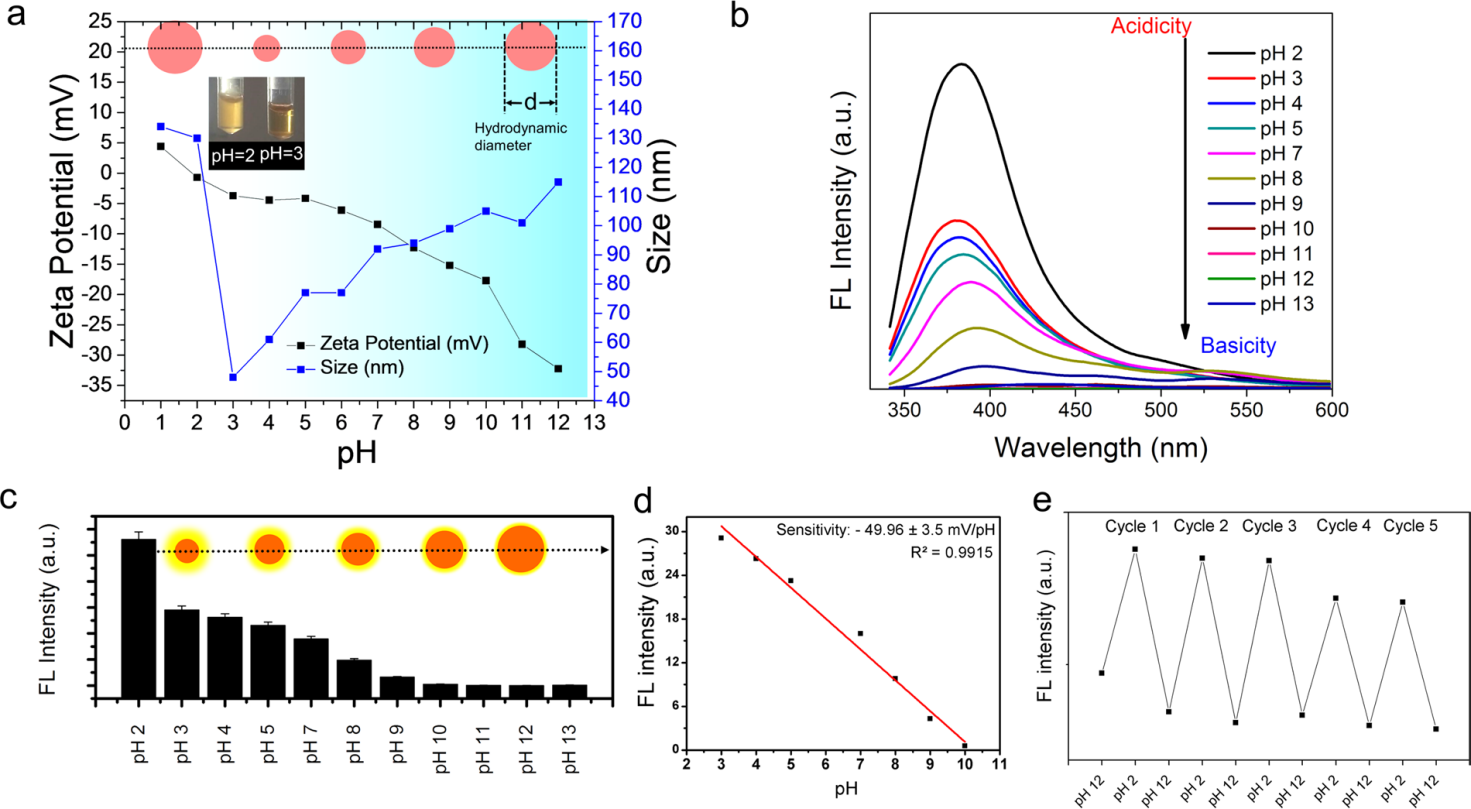
(**a**) Effect of pH on the zeta-potential and apparent size of the sGQDs Inset showing changes in the hydrodynamic diameter of sGQDs at various pH values and pictorial images of sGQDs at pH 2 and pH 3. (**b**) Decrease in the fluorescence intensity of the sGQDs with increasing the pH of the solution, (**c**) Fluorescence variation at 380 nm (inset shows the size of the sGQDs increases with increasing pH of the solution). (**d**) Linearity in the fluorescence intensity change between pH 3 and pH 10 with regression value (R^2^ = 0.9915). (**e**) Cyclic and reversible changes in the fluorescence intensity of the sGQDs on multiple cycles of pH variation (cycles between pH 2 and pH 10).

Size-dependent uptake of larger graphene oxide sheets (420 nm to 630 nm) have been reported earlier^6,7,46^, however, GQDs being much smaller fragments of GO could enter into the cells via multiple mechanisms. Briefly, the cell uptake occurs mainly via endocytosis mechanism. The endocytosis pathway is divided into two major pathways- phagocytosis and pinocytosis. The former involves uptake of relatively larger particles (micro-organism, viruses) and macromolecules which are actin-dependent, whereas the latter deals with the uptake of fluids and relatively smaller particles which may or may not be energy-dependent^47,48^. The pinocytosis mechanism is further divided into macropinocytosis, clathrin-mediated endocytosis, caveolin-mediated endocytosis, and clathrin (or caveolae) independent endocytosis. Clathrin-mediated endocytosis occurs via receptor-mediated endocytosis of particles followed by formation of clathrin-coated pits and recruitment of adaptor protein AP2 complex at the cell membrane^49^. Similarly, the caveolae-mediated endocytosis occurs at the lipid rafts supplied mainly by cholesterol in the cell membrane. The cytosolic protein, caveolin-1 binds to the cholesterol molecules to form invaginations and vesicles to cargo molecules inside the cell cytoplasm^50^. To study each of these uptake mechanisms and to elucidate the exact mechanism by which the sGQDs can enter the cell, specific cell uptake inhibitors are used. In this regard, we used various pharmacological cell uptake inhibitors^51–53^ to understand the uptake mechanism of the sGQDs in the L929 cells. The role of energy in the cellular uptake mechanism can be studied by using low temperature (4 °C), the metabolic activity remains in an inactive condition and hence the role of passive diffusion (energy independent) can be studied. Alternatively, the energy-dependent active process can also be checked by depleting the ATP molecules inside the cells at 37 °C. Sucrose solution at hypertonic conditions (0.5 M) is a potent inhibitor for clathrin-mediated endocytosis by disrupting the clathrin-coated pits and immobilizing adaptor proteins required to form vesicles at the plasma membrane^54,55^. The caveolae-mediated endocytosis can be blocked by nystatin that depletes the cholesterol level at the plasma membrane, thereby inhibiting attachment of the caveolin-1 protein to form caveosomes^48,51–53^. The cytochalasin D is mainly known for the depolymerization of actin-filaments and hence, can be used for blocking the actin-dependent phagocytosis mechanism^47,48,52^. Figure 9 shows CLSM imaging of cells treated with sGQDs at 4 °C or with cell uptake inhibitors (37 °C) such as NaN_3_, hypertonic sucrose, nystatin and cytochalasin D. Additionally, the toxicity analysis and optimum incubation period for cell treatment was determined prior to confocal imaging experiment (Supplementary Fig. S8). It can be seen that no apparent fluorescence was observed in the L929 cells labelled with the sGQDs at 4 °C and NaN_3_ suggesting that the primary mechanism of cell uptake is an active process (energy-dependent pathway). Similarly, low fluorescence was observed in cells treated with hypertonic sucrose and nystatin suggesting the role of clathrin- and caveolae-mediated endocytosis as the primary mechanism of the cell uptake for the sGQDs. Furthermore, high fluorescence intensity was observed in the cells treated with cytochalasin D negating the role of phagocytosis as the primary cell uptake mechanism for the sGQDs. To further confirm the cellular uptake mechanism, we performed a quantitative uptake study of the sGQDs using flow cytometry. Figure 10 shows the quantitative cell uptake of the sGQDs in the presence of various cell uptake inhibitors. In comparison to the untreated cells (Fig. 10a), ~95% sGQDs are taken up by the cells (Fig. 10b) within 8 h. However, the cells treated with sGQDs at 4 °C (Fig. 10c) and NaN_3_ (Fig. 10d) at 37 °C, merely show ~2% and ~15% cell uptake respectively. Hence, the uptake mechanism is primarily energy-dependent as low passive uptake was observed. Furthermore, cells co-incubated with sGQDs and hypertonic sucrose (0.5 M), or nystatin, which is clathrin and caveolae cell uptake inhibitors showed ~34% and ~16% cell uptake, respectively at 37 °C. Lower endocytosis of sGQDs in the presence of inhibitors suggests that the cell uptake followed primarily by a clathrin-and caveolae-mediated mechanism (Fig. 10e,f). Interestingly, cells co-incubated with sGQDs and cytochalasin D show ~92% cell uptake demonstrating that the phagocytosis does not form a primary mechanism of cell uptake of sGQDs (Fig. 10g). Previously, Wu *et al*. showed that the GQDs enter the cell via caveolae-mediated endocytosis mechanism and they localized inside the cell cytosol, even in the endoplasmic reticulum. Upon longer time incubation (3 days) the GQDs entered the cell nucleus but, changed the morphology of the cell which is usually not desired^56^. Quantitative cell uptake analysis shows that the cell uptake primarily occurred via clathrin- and caveolae-mediated endocytosis, and was an active energy-dependent process and less dependent on the passive process (Fig. 10h,i). Following cell internalization, the sGQDs efficiently and specifically self-localized into the nuclei of the cells. The sGQDs were not labeled with nuclear localizing signals or proteins. Previous reports on the fluorescent C-dots have shown that they can enter the cell nucleus because of its small size, surface charge and with an aid of histone-chaperones and nuclear-localization signals^57^. As we showed in Fig. 11, the size of the sGQDs decrease with a decrease in acidic pH, it is speculated that due to the variation of the pH in the endosomal compartments- early endosomes (pH 6.0 to pH 6.5) through late endosomes (pH 4.5 to pH 5.5)^58^, the size of the sGQDs decreased and thereby, rendering small-sized GQDs with negatively-charged surfaces which then entered the nucleus within 8 h.

Nuclear staining dyes (organic or peptide-based)^59^ are limited by poor photo-sensitivity, difficult to synthesize, and high cost. The sGQDs are biocompatible, label-free, self-localize inside the nucleus, and photostable for a long period. Therefore, we envisage sGQDs as an economical and environment-friendly alternative fabricated using a green carbon source for future nucleus staining dyes and also as a drug-delivery vehicle for nuclear targeting drugs due to their small surface-to-volume ratio and intrinsic fluorescence. Thus, sGQDs could be used for label-free selective intracellular localization inside cells without hampering the cell architecture and viability.

### Surface charge, size, effect of pH and photoluminescence pH sensing

The heteroatom-containing functional groups and the hydrophobic sp^2^ hybridized carbon skeleton in the sGQDs govern their surface charge and size. Lateral size analysis performed using dynamic light scattering (DLS) reveals that an average size of the sGQDs gets increased with increasing concentration of the sGQDs (Supplementary Fig. S9). The functional groups especially carboxylic, and phenolic groups not only contribute to their negative zeta potential of the sGQDs but also help in self-assembly process^60,61^. Figure 11a shows that size and surface charge of the sGQDs dramatically changes, size 45–130 nm, charge ca. + 4.43 to −32.2 mV, over the change in the pH. At neutral pH, an aqueous dispersion of the sGQDs show larger size than the sGQDs dispersed in ethanol (as previously illustrated in Fig. 2), due to hydrogen bonding between the functional groups of sGQDs and solvent^62^. It is now known that solvent plays a major role in the self-assembly process^63^. An average size of the sGQDs is ~70–90 nm at neutral pH (pH = 7.0). The TEM image showed slightly smaller diameter due to sample drop casting and drying over the copper grid. In the solution phase, the size significantly increases up to ~115 nm at basic pH (pH = 12.0) and considerably decreases to ~48 nm at acidic pH (pH = 3.0). It is worth noticing that there is a drastic increment in the size of the sGQDs (~130 nm) below pH 3, consistent with the previous reports^64–66^. At lower pH (<pH 3), protonation of the heteroatoms and related functional groups occurs leading to an increase in zeta potential and the hydrophobicity of the sp^2^ carbon atom containing surfaces inducing aggregation of the sGQDs (Supplementary Fig. S10). It is also clearly visible after adding few microliters of HCl into sGQDs dispersion (at <pH 3.0), the translucent dispersion of sGQDs becomes turbid indicating the agglomeration of sGQDs (Fig. 11a inset). The color of the sGQDs dispersion darkens from acidic to basic pH (Supplementary Fig. S11). On increasing the pH, deprotonation of the functional group reduces the zeta potential, and the electrostatic repulsion between the negatively charged functional groups prevent the aggregation of the sGQDs and stabilize them. Therefore, the size of the sGQDs does not significantly change between pH 7 and pH 11. However, after pH 11 the size of the sGQDs increases sharply due to salting effect^64,67^. The sGQDs exhibit a gradual decreasing pattern in the PL intensity over pH 2 to pH 13 (Fig. 11b,c). The sGQDs show high intense fluorescence emission at acidic pH, whereas at basic pH it is quenched. Wu *et al*. reported a reverse trend of PL property of the GQDs which stated that there are zigzag emissive sites (carbene-like) are present in the structure of the GQDs responsible for such behavior. These sites get protonated in acidic medium showing fluorescence quenching and restored fluorescence again in the alkali medium^68^. This indicates that our sGQDs do not possess carbene-like emissive sites in its structure and the defects are due to the presence of the functional groups. In literature, some reports show PL stability over the change in pH^69^ while some showed a decreasing trend in both acidic and alkali medium^70,71^. Basically, the fluorescence property of GQDs depends on the presence of conjugated π-domains and surface state which is closely related to hybridization of carbon skeleton, defects and functional groups attached to it^72^. The fluorescence of the sGQDs originated from the heteroatom containing functional groups which are present in ample amount over the surface of sGQDs which have been proved previously by the FTIR and XPS (Fig. 4). This corroborates with the previously shown report by Yan *et al*., showing fluorescence quenching at basic pH^73^. The protonation or deprotonation of the functional groups (-OH, >C=O, -COOH) at pH change affect the π-π* and n-π* transitions by refilling and depleting the valence band thereby quenching the fluorescence from the sGQDs.

We further evaluated the pH sensing capability of sGQDs against various pH range in aqueous solution. The quenching of the fluorescence emission characteristics of the sGQDs follows a linear relation (R^2^ = 0.9915) between pH 3 to pH 10 (Fig. 11d). Thus it provides a pH sensing window ranging from acidic to basic medium. For evaluation of sensing characteristics of sGQDs as pH sensing probes, we further analyzed various factors. The sGQDs showed good reversibility in the PL behavior on the cyclic pH variation analyzed between pH 2 and pH 12 for five consecutive cycles (Fig. 11e). The response time of each pH sensing fluorescence was found to be less than 1 min, and the sensitivity was calculated to be −49.96 ± 3.5 mV/pH units at 29 °C. The additional advantage of the sGQDs was that they were highly dispersible in the sensing pH range (Supplementary Fig. S11).

The sGQDs owing to its stable fluorescence characteristics and rapid response time can be used for the toxic metal ions or analyte detection techniques for point-of-care applications^74^. Furthermore, owing to its red-luminescence emission property, they can also be applied for non-invasive analyte detection inside cells^75,76^. Thus, due to nucleus targeting ability and fluorescence sensing capability, we envisage potential applications of sGQDs over a wide field of research in theranostic, drug delivery, biosensors, and biomedical nanotechnology.

## Conclusions

In conclusion, a facile one-pot green synthesis approach was used to fabricate sGQDs for biomedical applications. Synthesis and purification are quite simple, scalable and do not employ any hazardous reagent during the process. Synthesized sGQDs are bright fluorescent, water dispersible and show self-assembly in aqueous solution. The sGQDs showed cell proliferating activity in fibroblasts and selective nucleus labelling in mammalian cells. It was observed that the sGQDs enter the cells via energy-dependent clathrin- and caveolae-mediated endocytosis pathway followed by localizing themselves inside the cell nucleus. Thus, it can form a nucleus-targeting drug delivery vehicle accompanied by its inherent fluorescence property in theranostic applications. As a labelling agent, these sGQDs can be an economical alternative to existing nucleus labelling agents due to their cytocompatibility, photostability and near-infrared fluorescence emission paving the way for non-invasive pH detection opportunities. Selective labelling of cellular organelles is of particular importance to investigate the cellular mechanisms in different parts of the cell. Intracellular pH mapping could pave the way for better understanding of the various physiological phenomenon that occurs in a microenvironment of the cell in molecular diagnostics and development of cost-effective alternative sensing probes.

## Methods

### Instrumentation

The UV-Vis spectroscopy was performed using Perkin Elmer Lamda-25. Fluorescence spectroscopy was performed using Shimadzu at a slit width of 5 nm (excitation and emission) at high sensitivity mode. Morphological studies were performed using Field-Emission Gun Transmission Electron Microscopy (FEG-TEM), High-resolution Transmission Electron Microscopy (HR-TEM) Philips CM200, and cryo-TEM (JEOL). The Fourier Transform Infrared spectroscopy (FTIR) was done using 3000 Hyperion Microscope with Vertex 80 FTIR System (Bruker, Germany). X-ray diffraction (XRD) was performed using PANalytical -XRD. Raman spectra were recorded using a Jobin-Yvon Labram spectrometer. X-ray Photoelectron Spectroscopy (XPS) was performed using a monochromatic Al K-alpha source (225 W) with a Kratos Analytical, UK (Model AXIS Supra). Fluorescence microscopy was performed using Inverted Fluorescent Microscope: Nikon Eclipse TE 2000-S. Confocal laser scanning microscopy (CLSM) was performed using (Olympus, Flow view). The size and zeta-potential measurements at different pH values were performed using Malvern Zetasizer.

### One-pot microwave-assisted synthesis of self-assembled GQDs

Grape seed extract (Dietary supplement) procured from Zenith Nutrition, USA. Absolute ethyl alcohol (AR grade, 99.9%) was procured from Jiangsu Huaxi International Trade Co., Ltd. China. Briefly, GSE powder (17.0 g) was dissolved in absolute ethanol (50 mL), and it was centrifuged at 2000 rpm for 10 min. The supernatant was stored, and its solvent was evaporated. The residue was again dispersed in milli-Q water and was heated under Microwave oven until all liquid part was eliminated. Residual solid was again dispersed in absolute ethanol, and it was centrifuged at 2000 rpm for 10 minutes. The supernatant was filtered through 0.45 µm pore sized syringe filter. Dispersed and purified liquid content was dried using rotary evaporator and hot-air oven to yield of solid powdered GQDs (805.0 mg). Obtained solid was dispersed in ethanol and milli-Q water separately, and their fluorescence absorbance study was done. Ethanolic and water dispersion of GQDs were imaged under JEOL 2100 Field Emission Gun Transmission Electron Microscopy (FEG-TEM). The GQDs were also characterized using FTIR, Raman, XPS, UV-visible and fluorescence spectroscopy.

### Cytotoxicity and cell proliferation studies

For biocompatibility assay, L929 cells were treated with GSE and sGQDs at different concentrations (5–100 µg/mL) for 24 h. Cell viability was measured using MTT assay. To analyze the effect of the proliferation-inducing capacity of sGQDs, an *in vitro* scratch wound assay was performed^77^. A scratch was made using a sterile-tip making a wound on confluent adherent cultured cells in each well of 12-well plates. Cells were then washed and re-incubated with and without sGQDs. Cells were imaged at regular time intervals till the wound was completely closed. Comparative scratch wound closure was calculated as percentage wound area using Image J as a function of different time intervals. Also, the cell proliferation (with or without treatment) was studied using a hemocytometer. The L929 cells were trypsinized after 24 h, 48 h, and 72 h after treatment with sGQDs. The cells were loaded into the counting chamber (around 25–30 cells/mm^2^) for cell counting. Untreated cells were considered as control cells.

### Cell cycle analysis and reactive oxygen species (ROS) determination using flow cytometry

To investigate the effect of sGQDs on the cell proliferation, cell cycle analysis was performed. Briefly, cells were incubated with sGQDs for various time intervals (2 h, 4 h, 6 h, 8 h, 12 h) followed by cell harvesting, and fixation with chilled ethanol. After some washing steps (2–3 min), the cells were redispersed in propidium iodide (PI, 40 µg/mL) and RNase (100 µg/mL) in PBS (pH 7) and cell cycle was recorded using flow cytometry. To assess the role of ROS in the cell proliferation rate, the ROS production was determined using 2′, 7′-dichlorodihydrofluorescein diacetate (H_2_DCF-DA, Sigma-Aldrich). Untreated cells were regarded as negative control and cells treated with hydrogen peroxide (50 µM) were considered as positive control. Briefly, the sGQDs or GSE treated cells were treated with H_2_DCF-DA (10 µM) and kept on ice till the flow cytometry readings were recorded under FITC filter. For all flow cytometry experiments, a minimum of 10,000 events was recorded, and the data were processed using FlowJo V10 package.

### Confocal laser scanning imaging and fluorescence-tracking experiments

Fibroblast L929 cells were seeded on sterile cover-slips in a 12-well plate at a concentration of 8000 cells/well. Sterile sGQDs (100 µg/mL) were added, and the cells were incubated at 37 °C at 5% CO_2_ for the different period (2 h, 4 h, 6 h, 8 h). The fluorescence emission and co-localization of the sGQDs (from cytoplasm as well as the nucleus) were compared to the fluorescence emission from DAPI (labelling nucleus) with time to understand the internalization of sGQDs from cytosol to the inside of the nucleus. Similarly, other cell lines such as HT-1080, MIA PaCa-2, HeLa, and MG-63 cells were also treated with sGQDs for nucleus imaging and quantitative analysis and stained by DAPI for comparison. For each experiment, the cells were fixed with formaldehyde solution (3.7%) for 15 min followed by washing thrice with PBS. The fixed cells were stained with DAPI (2.0 µg/mL), incubated for 15 min followed by three cycles of PBS washing. The cells were kept for 5 min in the PBS during washing cycles. The CLSM imaging was performed by using multi-photon excitation for DAPI blue filter (750 nm) and the sGQDs, red filter (560 nm).

## Cell uptake mechanism analysis

The L929 cells were seeded in a six-well plate with complete media for 12 h cultured at 37 °C in 5% CO_2_ in Dulbecco’s Modified Eagle’s Medium (DMEM) supplemented with foetal bovine serum (FBS, 10%) and 1% penicillin/streptomycin (PenStrep, 1%). After cell attachment, the growth medium was replaced with serum-free media and inhibitors (at 37 °C) or at 4 °C for 1 h incubation. The inhibitors include hypertonic sucrose (0.5 M), nystatin (20 µg/mL), NaN_3_ (0.1%), cytochalasin D (5 µg/mL). Following incubation, the cell medium was replaced with serum-free growth medium containing sGQDs (100 µg/mL) and inhibitors (at 37 °C) or 4 °C for 8 h incubation. Cells were also washed thrice with PBS (pH 7) and fixed using formaldehyde (3.7%, pH 7). The cells were stained with DAPI (2 µg/mL) and analyzed using CLSM. Similarly, for quantitative cell uptake, the cells were co-incubated with sGQDs and cell uptake inhibitors (at 37 °C) or at 4 °C for 8 h. The cells were washed thrice, trypsinized and resuspended in PBS (pH 7). A minimum of 10,000 events was recorded and the data was processed using FlowJo V10 package.

### Optical pH sensing and size/charge measurements

Briefly, 100 µg/mL sGQDs were adjusted to different pH values using HCl (1.0 M) or NaOH (1.0 M) for sensitivity and reversibility experiments. Photoluminescence study was performed at excitation wavelength 320 nm, and its emission was analyzed at different pH values. The cyclic switching in the pH values between pH 2 and pH 12 was done by acid-base titration using HCl (1 M) or NaOH (1 M). For fluorescent measurements, the excitation and emission slit widths parameters were kept constant throughout the recordings.

### Data Availability

All data generated or analysed during this study are included in this published article (and its Supplementary Information files).

## Electronic supplementary material

Supplementary information
