# Supplementary material for: Graphene Quantum Dots for Cell Proliferation, Nucleus Imaging, and Photoluminescent Sensing Applications
Source: Sci Rep. 2017 Nov 20;7:15858. doi: 10.1038/s41598-017-16025-w (PMC5696518; doi:10.1038/s41598-017-16025-w)
Supplement: Supplementary file 1 — Supplementary information [file 41598_2017_16025_MOESM1_ESM.pdf]

Supplementary Information (SI) for

Graphene Quantum Dots for Cell Proliferation, Nuclear Imaging, and Photoluminescent Sensing Applications

Mukesh Kumar Kumawat, Mukeshchand Thakur, Raju B. Gurung, Rohit Srivastava*

Department of Biosciences and Bioengineering, Indian Institute of Technology Bombay, Mumbai- 400076, Maharashtra, India.

Department of Biosciences and Bioengineering, Indian Institute of Technology-Bombay, Powai, Mumbai-400076, India.

***Corresponding author: Prof. Rohit Srivastava, Phone: +91-022-25764761,**

Email: rsrivasta@iitb.ac.in, Tel: +91 22 25767746.

Scheme S1 Quantum yield (QY) calculation:

QY was calculated using quinine sulfate (QY:54%) as a reference. Briefly, QS was dissolved in 1 M H_2SO_4 (η:1.33), and sGQDs were dissolved in Milli-Q (η 1.33). The QY was calculated using following equation 1:

$$\Phi_{sGQDs} = \Phi_{QS} \, {.I_{GQDs} . \eta_{GQDs}{}^{2}} \big/ {.I_{QS} . \eta_{QS}{}^{2}} \qquad \text{(Equation 1)}$$

Where, ϕ is the QY, I is the gradient of integrated PL, η is the refractive index of solvent in which GQDs and QS were dispersed. The QY calculated was 31.79%.

**Sample**	**Gradient of integrated PL spectra**	**η**	**Q.Y. (%)**
Q.S.	1633.7	1.33	54%
sGQDs	961.82	1.33	**31.79%**

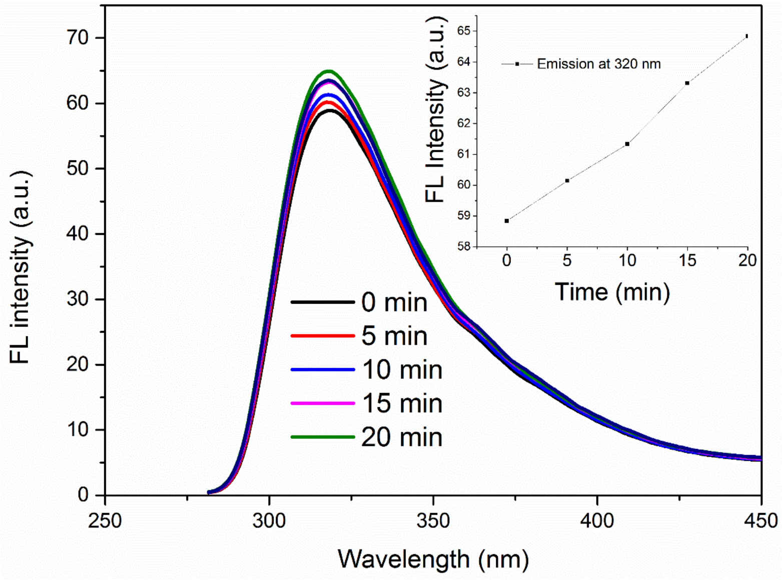

Figure S1. The increase in the FL intensity of sGQDs after ultrasonication at different time intervals. Inset showing a linear rise in the emission maximum at 320 nm.

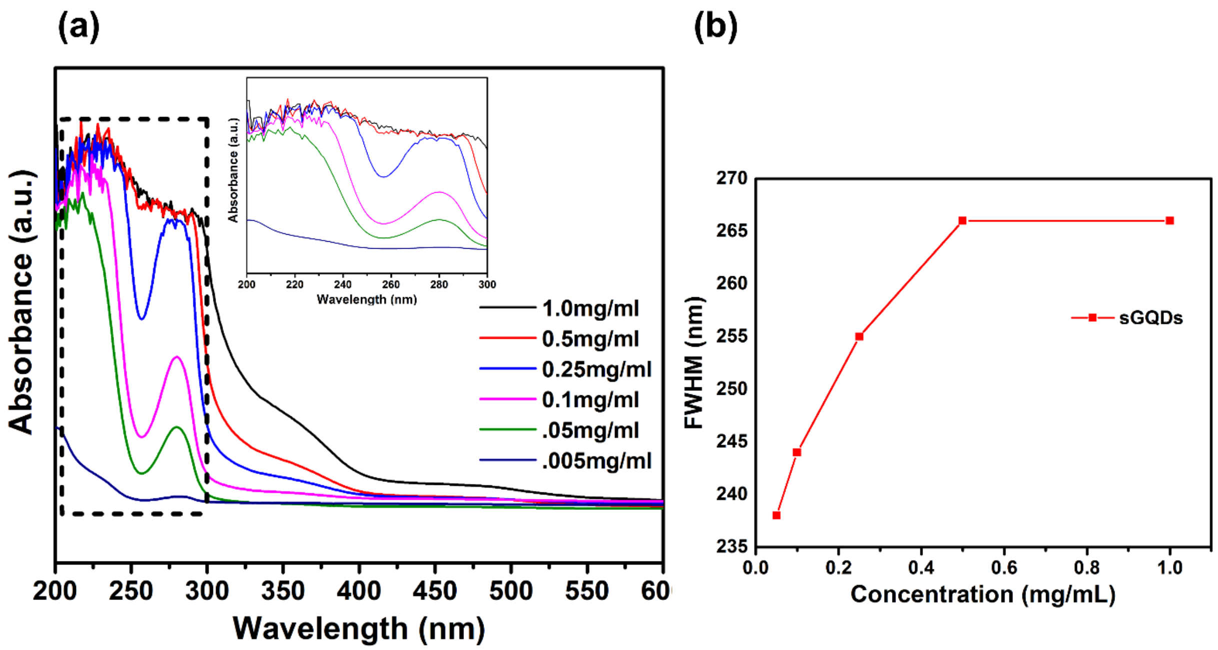

Figure S2. A UV-visible absorbance study of the sGQDs in aqueous solution at different concentrations. (a) Absorption spectrum shows that the peak gets broadened with increment in the concentration (inset). (b) The FWHM increases with increase in the concentration and then reaches towards saturation.

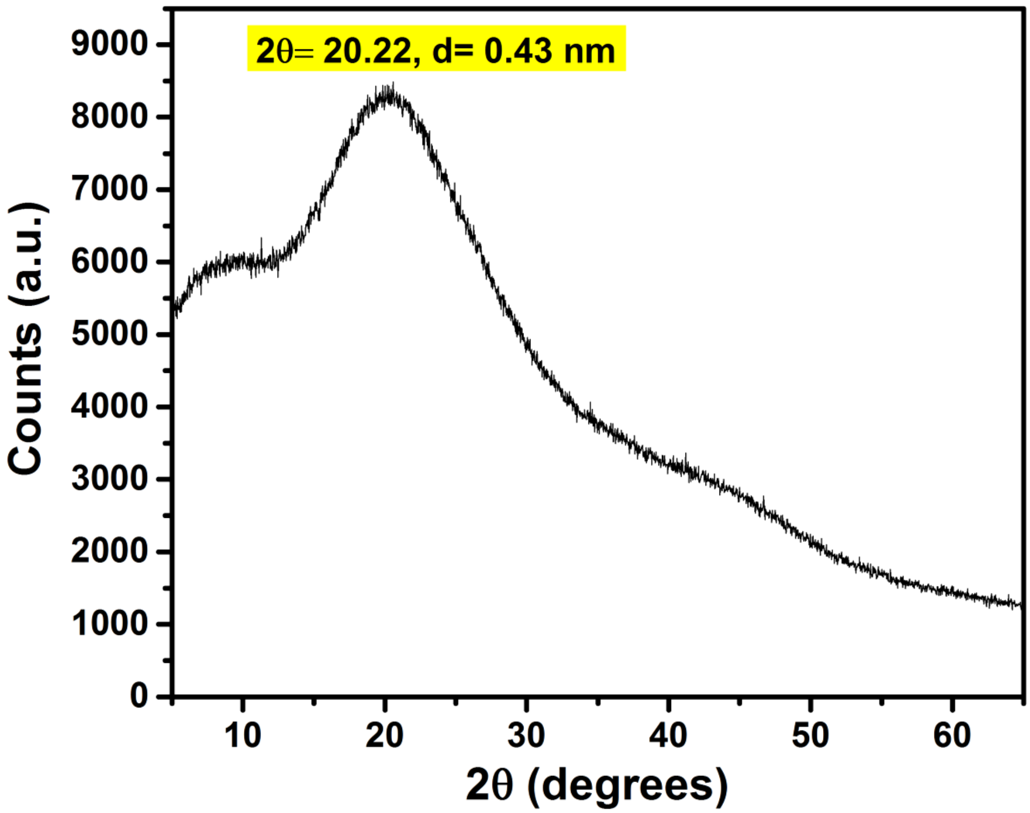

Figure S3. X-ray diffraction pattern of sGQDs powder. The 2θ=20.225° is equal to interlayer distance 0.438 nm which is greater than the interlayer distance of graphite (0.34 nm) indicating the incorporation of functional groups on the surface.

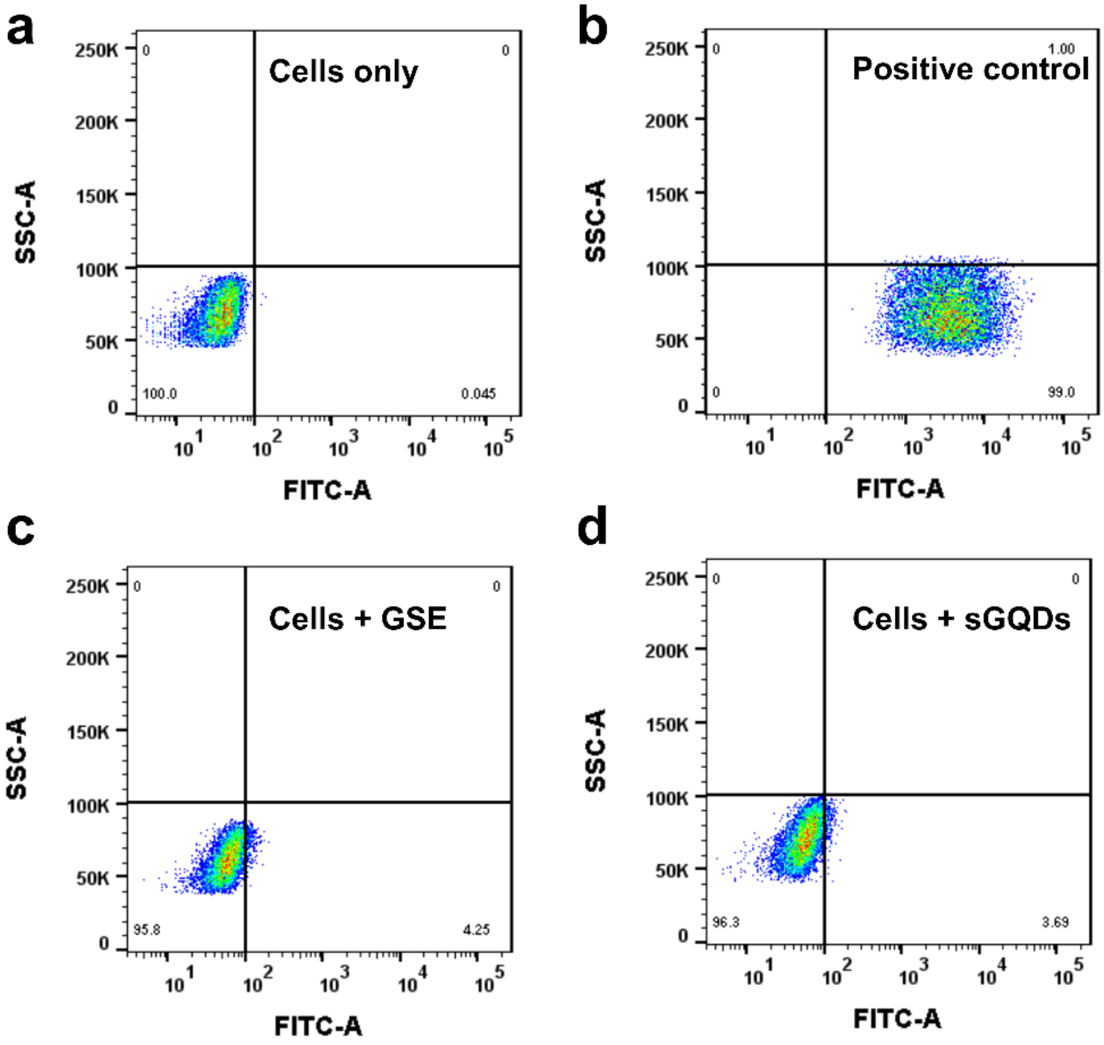

Figure S4. The ROS production and their effect on L929 cells treated with sGQDs. (a) untreated L929 cells. Cells treated with (b) H_2O_2 , (c) GSE and, (d) sGQDs.

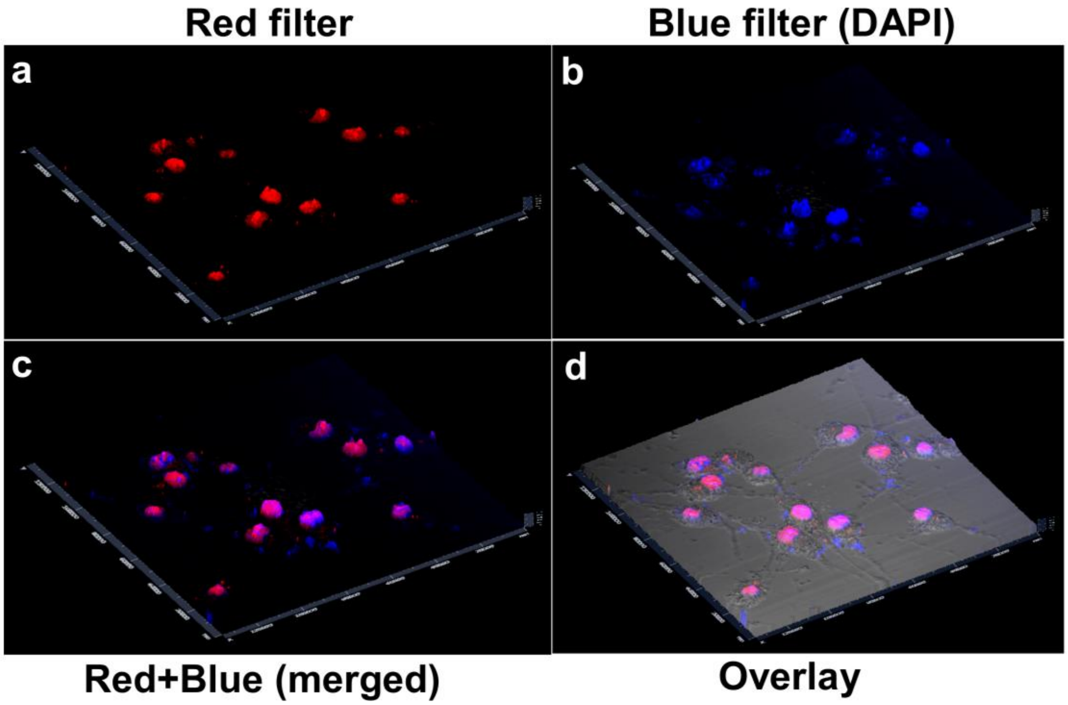

Figure S5. A 2.5 D view of a region of L929 cells under confocal microscope indicating the co-localization of sGQDs and DAPI in the nuclei of the L929 cells after 8 h.

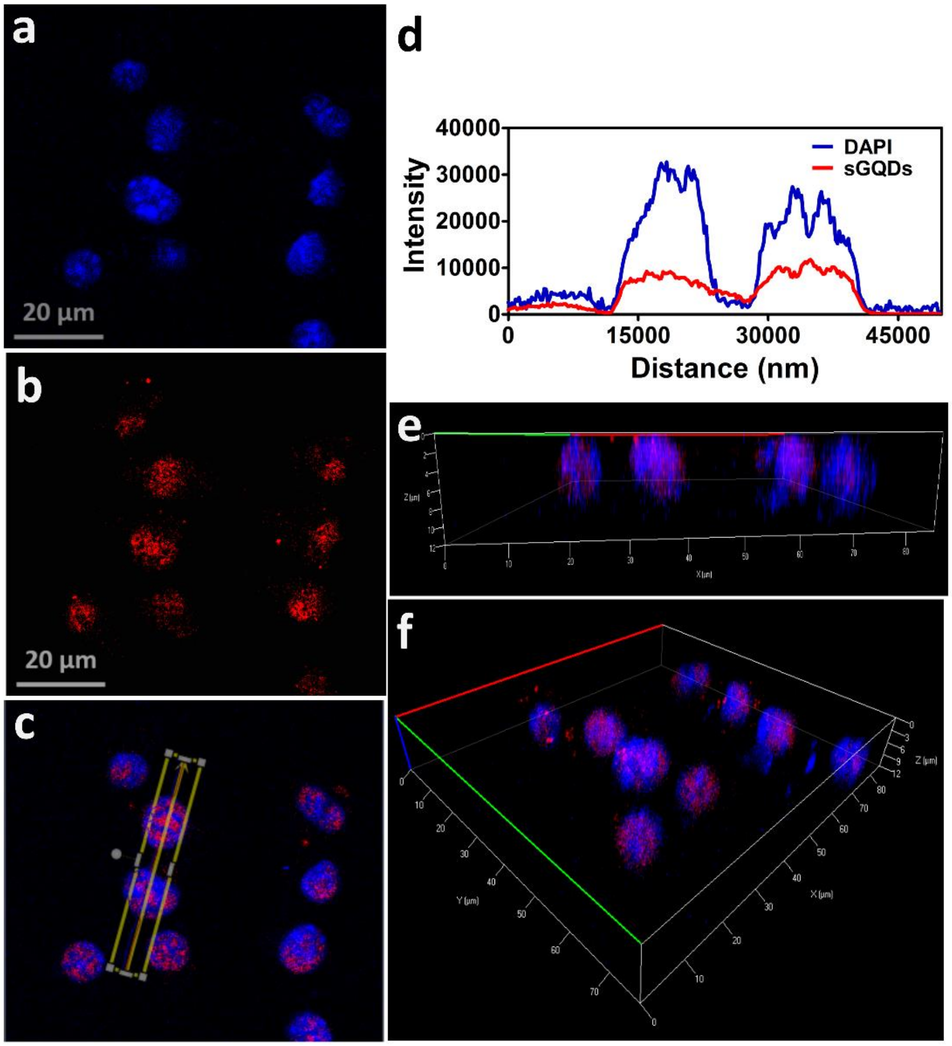

Figure S6. Intra-nuclear localization and quantification of sGQDs inside L929 cells using 3D-CLSM imaging. Cells labelled with DAPI (a) and sGQDs (b) after 8 h show co-localization inside the cell nucleus (c). (d) A comparison of fluorescence quantification of the sGQDs with DAPI across the scanning line (shown in (c)). A 3D representation of sGQDs with DAPI (e-f) show intra-nuclear co-localisation.

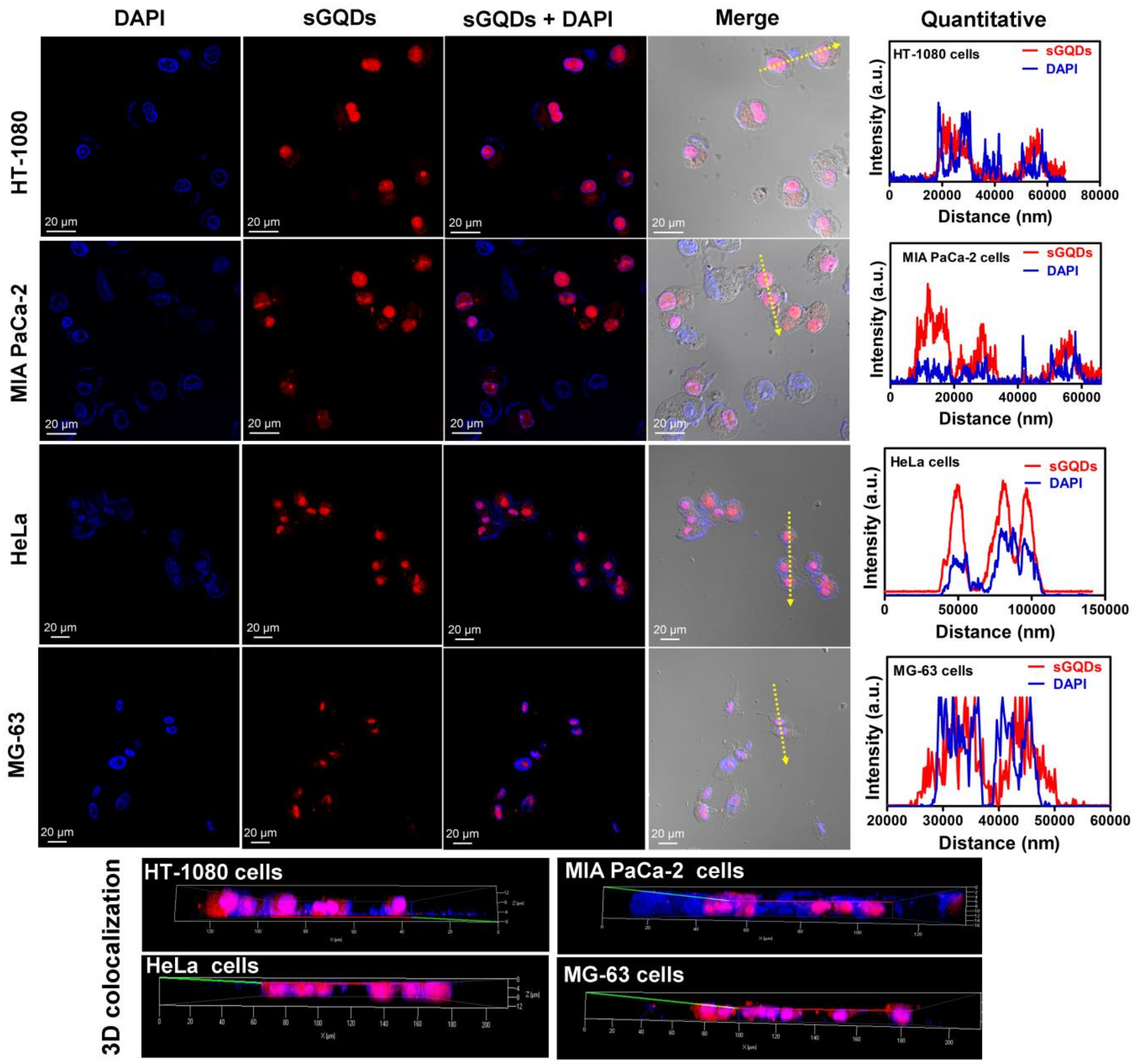

Figure S7. Different cells showing nucleus labelling using sGQDs. The sGQDs co-localize with DAPI within 8 h incubation at 37°C. The 2D co-localization of DAPI and sGQDs show clear quantitative uptake (right panel) and the 3D co-localization show intracellular localization in all the cells (lower panel).

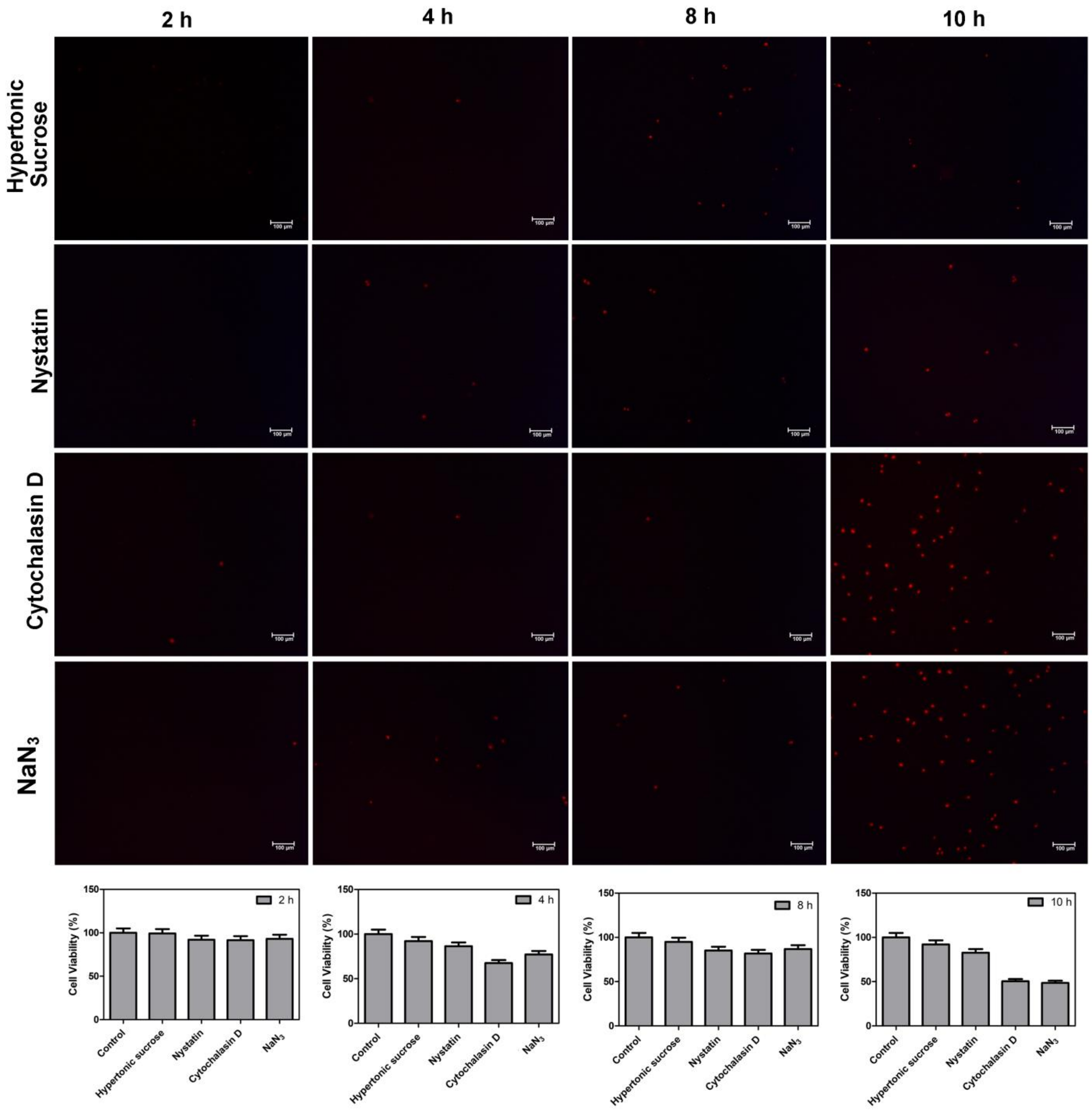

Figure S8. Time-dependent cell cytotoxicity of cell uptake inhibitors- hypertonic sucrose (0.5 M), nystatin (20 μg/mL), cytochalasin D (5 μg/mL), and NaN_3 (0.1 %) on L929 cells at 37°C. Upper panel shows qualitative cell cytotoxicity analysis of L929 cells treated with cell uptake inhibitors using propidium iodide (10 μg/mL) staining method for 15 min under a fluorescence microscope (Ex/Em: 550/580 nm). Lower panel shows

corresponding quantitative cell cytotoxicity analysis using Alamar blue assay at each time period (2 h, 4 h, 8 h, and 10 h), n=3 and results are shown as mean±SD.

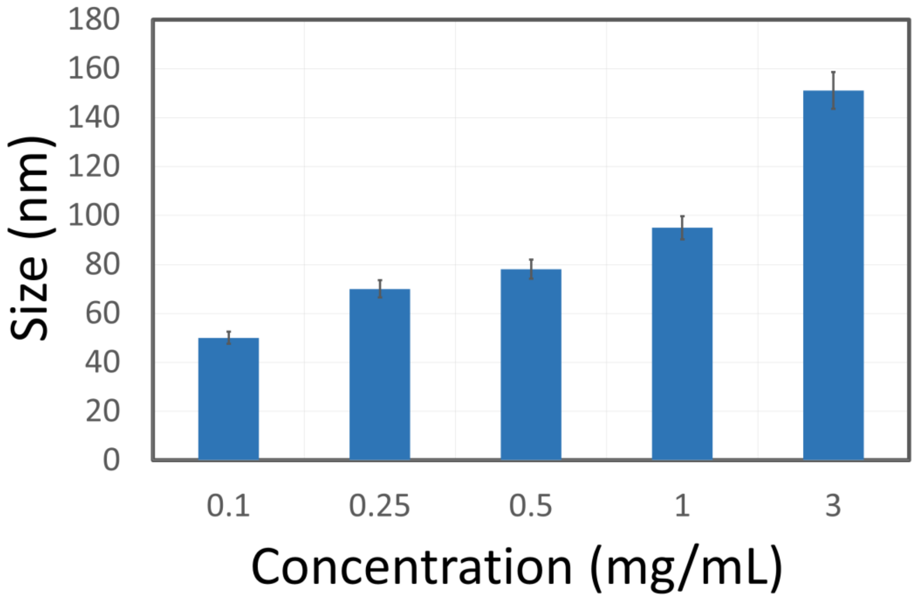

Figure S9. Hydrodynamic diameter analysis of sGQDs at different concentrations. The size of the sGQDs increases on increasing the concentration.

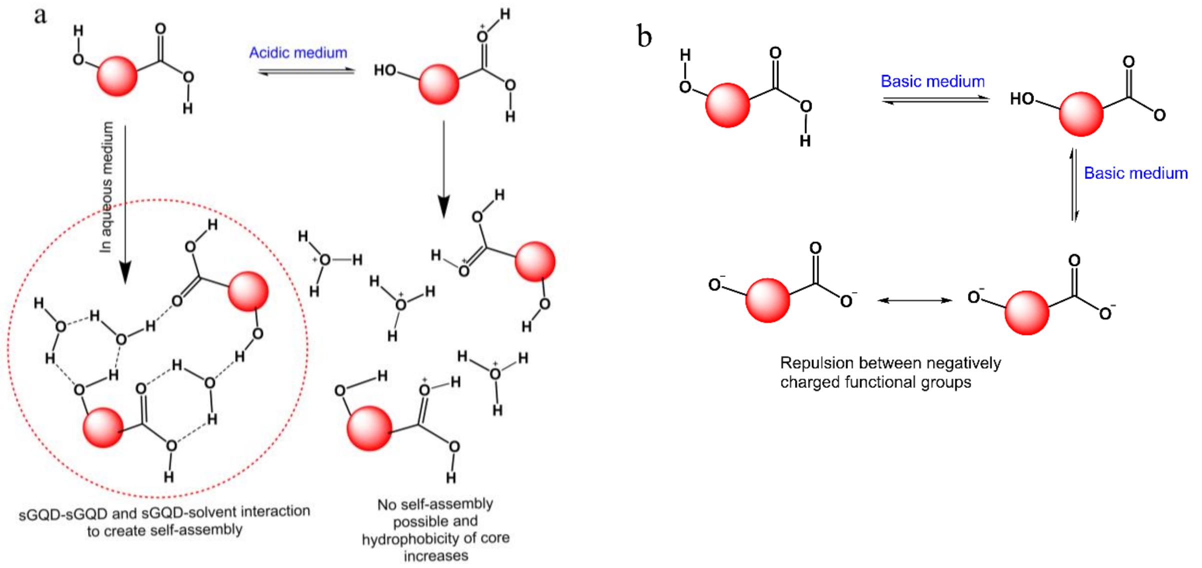

Figure S10. Choice of the solvent and pH affects the self-assembling of the sGQDs and hence affects the size and charge over their surfaces. Water molecules aid in the self-assembly process. (a) In acidic medium these functional groups are protonated and hydronium ions are formed from water molecules, hence, sGQDs-sGQDs, sGQDs-solvent interactions diminishes which increases the hydrophobicity of the core of the sGQDs encourages agglomeration (if pH is less than 3.0). (b) In the basic medium, hydroxyl ion arrest the protons from the carboxylic functional groups and carboxylate ions are formed. The repulsion between negatively charged ions occur and stabilize the sGQDs in the solution.

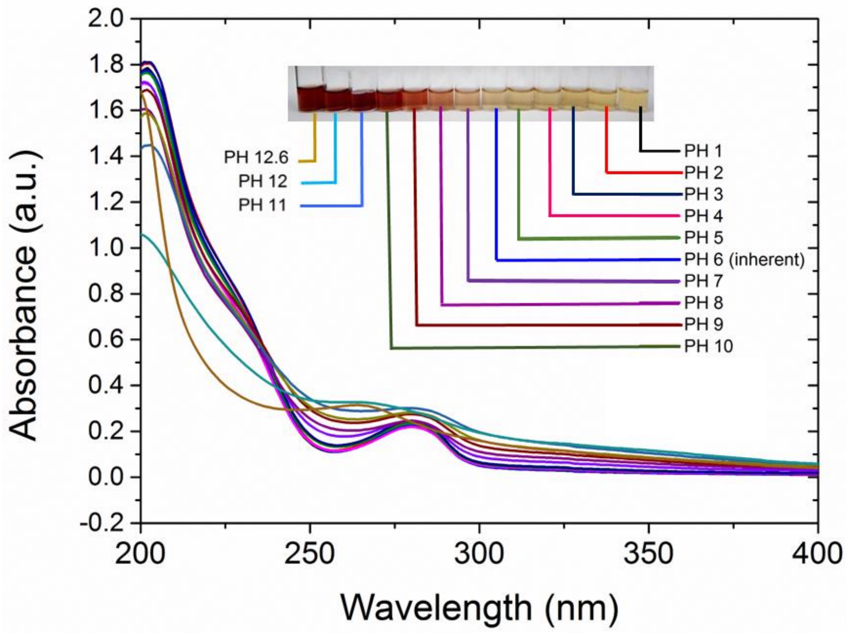

Figure S11. The absorption spectrum of sGQDs at different pH values. Inset is showing their corresponding light images of the sGQDs solution.
